# DRUG-seq for miniaturized high-throughput transcriptome profiling in drug discovery

**DOI:** 10.1038/s41467-018-06500-x

**Published:** 2018-10-17

**Authors:** Chaoyang Ye, Daniel J. Ho, Marilisa Neri, Chian Yang, Tripti Kulkarni, Ranjit Randhawa, Martin Henault, Nadezda Mostacci, Pierre Farmer, Steffen Renner, Robert Ihry, Leandra Mansur, Caroline Gubser Keller, Gregory McAllister, Marc Hild, Jeremy Jenkins, Ajamete Kaykas

**Affiliations:** 1 Neuroscience Research, Novartis Institutes for Biomedical Research, 250 Massachusetts, Cambridge, MA 02139 USA; 2 Chemical Biology & Therapeutics Informatics, Novartis Institutes for Biomedical Research, Fabrikstrasse 22, 4056 Basel, Switzerland; 3 Chemical Biology & Therapeutics, Novartis Institutes for Biomedical Research, 250 Massachusetts, Cambridge, MA 02139 USA; 4 Scientific Computing, Novartis Institutes for Biomedical Research, 250 Massachusetts, Cambridge, MA 02139 USA; 5 Analytical Sciences & Imaging, Novartis Institutes for Biomedical Research, 250 Massachusetts, Cambridge, MA 02139 USA; 6 Chemical Biology & Therapeutics Informatics, Novartis Institutes for Biomedical Research, 250 Massachusetts, Cambridge, MA 02139 USA; 7 Present Address: Blueprint Medicines, 45 Sidney St, Cambridge, MA 02139 USA

## Abstract

Here we report Digital RNA with pertUrbation of Genes (DRUG-seq), a high-throughput platform for drug discovery. Pharmaceutical discovery relies on high-throughput screening, yet current platforms have limited readouts. RNA-seq is a powerful tool to investigate drug effects using transcriptome changes as a proxy, yet standard library construction is costly. DRUG-seq captures transcriptional changes detected in standard RNA-seq at 1/100^th^ the cost. In proof-of-concept experiments profiling 433 compounds across 8 doses, transcription profiles generated from DRUG-seq successfully grouped compounds into functional clusters by mechanism of actions (MoAs) based on their intended targets. Perturbation differences reflected in transcriptome changes were detected for compounds engaging the same target, demonstrating the value of using DRUG-seq for understanding on and off-target activities. We demonstrate DRUG-seq captures common mechanisms, as well as differences between compound treatment and CRISPR on the same target. DRUG-seq provides a powerful tool for comprehensive transcriptome readout in a high-throughput screening environment.

## Abstract (web summary)

RNA-seq is a powerful tool to investigate how drugs affect the transcriptome but library construction can be costly. Here the authors introduce DRUG-seq, an automated platform for high-throughput transcriptome profiling.

## Introduction

High-throughput screening has been a staple in drug discovery over the past four decades^1^. Target-based drug discovery relies heavily on singular readouts such as reporter gene expression or modification of enzymatic activity in response to small molecule treatment. However, with a recent renewed focus on phenotypic based drug discovery^2^ there is an increased interest in more comprehensive and less-biased screening methods that combine aspects of both target-based and phenotypic screening, such as RNA-seq. However, there is a need to develop RNA-seq methods that are higher throughput and have reduced cost, so that it becomes feasible to screen large sets of compounds under multiple experimental conditions.

For that purpose, multiple transcriptional profiling platforms have been developed. Targeted sequencing-based approaches, such as RASL-seq^3^, measure up to a few hundred specific genes or splicing events. RASL-seq is particularly useful for studying genes of interest or genomic loci, where a focused panel of events can be assessed^3^. The Luminex L1000 platform, used for the Connectivity Map (CMAP), measures a fixed panel of about 1000 landmark genes and about half of the additional genes in the transcriptome are imputed in silico^4,5^. The latest release of CMAP provides a huge collection of phenotypic data of various perturbagens including compounds, shRNA and cDNA^5^, and has facilitated identification of small molecule leads for various disease studies^6,7^. L1000 has provided a very useful and cost-effective platform for transcriptional profiling. However, it currently only measures around 1000 genes and relies on imputation of the remaining genes instead of direct measurement^5^.

Whole transcriptome RNA-seq has become an attractive option to allow deeper interrogation of complex changes, yet most of the standard protocols are labor intensive and cost prohibitive for high-throughput use. The latest development, PLATE-seq, allows samples in 96 wells to be profiled at about $15 per sample^8^. However, it requires lengthy RNA purification steps with special oligo(dT) grafted plates, and at 96 well format, severely limits the number of treatment conditions that can be tested in a single experiment (Supplementary Fig. 1). It would be ideal to have a cost effective, massively parallelized transcriptome profiling method in 384- and 1536-well format to measure all genes in an unbiased manner to fully capture the transcriptional diversity induced by compound or genetic perturbation for drug discovery.

## Results

### DRUG-seq is a cost effective digital transcriptional profiling method used for high-throughput profiling

**Fig. 1:**
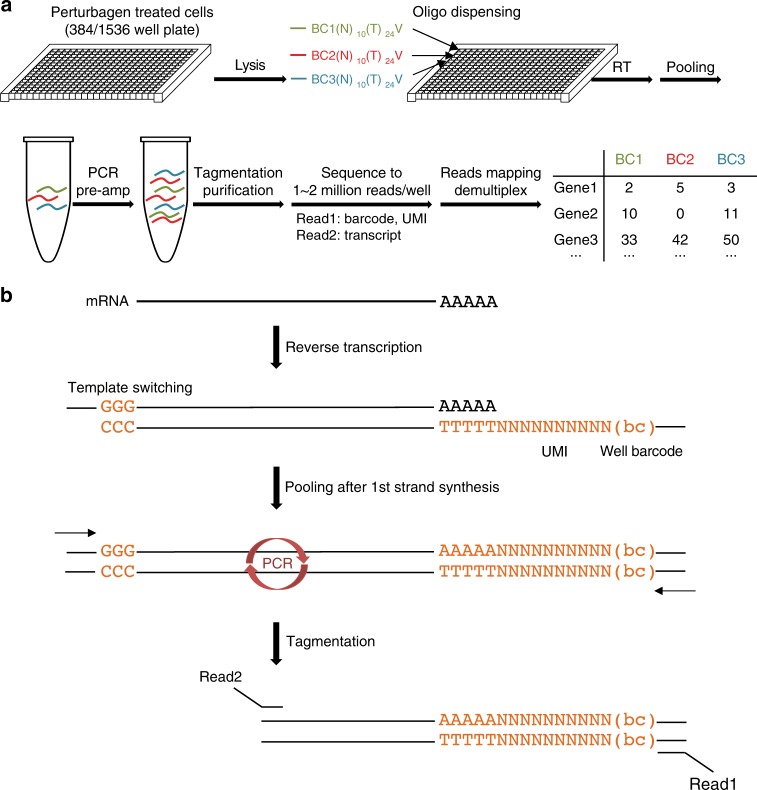
DRUG-seq overview. **a** DRUG-seq workflow. See details in Methods. Following compound treatment, cell lysis and RT reaction assembly are carried out with automation. Incubation steps are carried out in 384-well thermocyclers (Biorad C1000 Touch). All sequencing reaction was performed on Illumina Hiseq 4000 or Nextseq 500 platforms. **b** DRUG-seq chemistry. After cell lysis, mRNAs are directly reverse transcribed by a modified poly(dT) primer, which contains a well position specific 10mer barcode and a random 10mer sequence as unique molecular index (UMI). Template switching activity of the RT enzyme adds oligo(dC) to the first-strand cDNA, which allows binding of the template switching oligo (TSO). Samples are pooled after the RT and template switching. After pre-amplification and tagmentation, paired end libraries are sequenced to identify well position, UMI and transcript information

We developed a cost-effective method with increased throughput termed (DRUG-seq), which costs $2–4 per sample including sequencing expense and enables profiling in both 384- and 1536-well formats. By forgoing RNA purification and employing a multiplexing strategy, DRUG-seq simplifies multi-well processing to direct lysis and RT reaction steps and drastically cuts down library construction time and costs (currently $0.9 per well for 384-well plate and $0.2 for 1536-well plate). Furthermore, DRUG-seq allows the complete operation to be integrated with high-throughput automation (Fig. 1a). By incorporating specific barcodes into RT primers, cDNAs from individual wells are labeled and then pooled after first-strand synthesis, significantly reducing labor involved in multi-well processing. The template switching property of reverse transcriptase adds poly(dC) after first-strand cDNA synthesis and allows the binding of a template switching oligo (TSO) for pre-amplification by PCR. Following tagmentation and amplification, libraries are size-selected and sequenced with custom primers. RT primers also contain a 10 nucleotide Unique Molecular Index (UMI)^9^ to monitor potential PCR amplification artifacts (Fig. 1a, b, see details in Methods and Supplementary Methods).

To assess potential well-to-well cross contamination during sample processing, in a mixed species pilot experiment, we interweaved mouse origin C2C12 and human origin 293 T cells in the same 384 well plate and pooled material after indexed RT reaction (Supplementary Fig. 2a). After sequencing and read mapping, more than 98% of the wells have > 96% species specific UMI. Samples were separated by species in hierarchical clustering, and wells from the same species strongly correlate with each other, suggesting high reproducibility among wells and very little cross contamination during sample processing (Supplementary Fig. 2b and 2c).

**Fig. 2:**
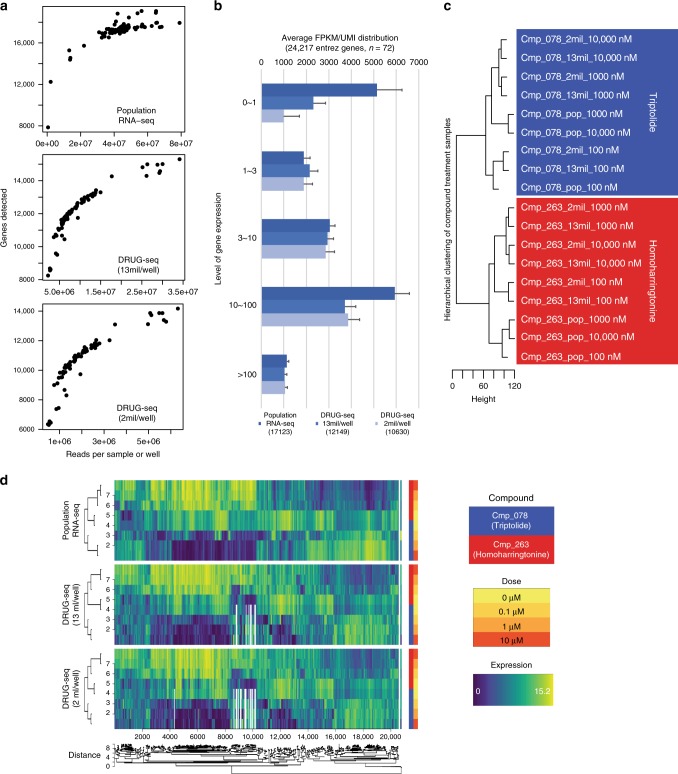
DRUG-seq performance is on par to standard population RNA-seq. **a** Gene detection distribution in population RNA-seq and DRUG-seq sequenced at 13 mil/well and 2 mil reads/well. **b** Gene detection comparison between population RNA-seq, DRUG-seq sequenced at 13 mil/well and 2 mil reads/well. Mean number of genes detected with standard deviation was broken down into different levels, *n* = 72 for each platform and read depth. **c** Differential gene expression detected by population RNA-seq and DRUG-seq both distinctively cluster samples treated with different compounds. Pop: population RNA-seq. 13 mil: DRUG-seq sequenced average at 13 mil reads/well. 2 mil: DRUG-seq sequenced at 2 mil reads/well. **d** Compound impact on differential gene expression is captured by both population RNA-seq and DRUG-seq in dose-dependent manner in meta-hierarchical clustering. The dendrogram located under the heatmaps represent the average correlation between clusters of the tree branch. See details in Methods

DRUG-seq is digital and counts the 3′ end of transcripts with the potential to reduce read depth and sequencing cost relative to standard RNA-seq. We tested the effect of reducing the read depth on the number of genes detected as well as on the accuracy of capturing differentially expressed genes under compound perturbation. A potent transcription inhibitor Cmp_078 (triptolide) and a translation inhibitor Cmp_263 (homoharringtonine) were chosen to provide different MoA for benchmarking. Samples were treated in triplicates either with DMSO or increasing doses of compounds at 0.1 μM, 1 μM, or 10 μM. DRUG-seq libraries were sequenced at estimated 2 million reads/well and 13 million reads/well and compared with standard population RNA-seq sequenced at an average of 42 million reads per sample. Compared with a median 17 K entrez genes detected in population RNA-seq, DRUG-seq detected a median of 11 K genes at 2 mil reads/well and 12 K genes at 13 mil reads/well (Fig. 2a), including most of the L1000 landmark genes (Supplementary Fig. 3a). Even at the shallow read depth of 2 mil reads/well, gene expression was highly consistent across wells (Supplementary Fig. 4a and 4b). A common concern shared by all 3′ counting platforms is the misquantification of pseudogenes. However, we did not observe significantly skewed quantification of pseudogenes in the DRUG-seq platform compared with population RNA-seq (Supplementary Fig. 4c). The majority of gains from population RNA-seq compared to DRUG-seq derives from lowly expressed genes with FPKM between 0 and 1, indicating recovery of low abundance transcripts is limited by starting materials. Comparing the performance of 2 and 13 million read depths per well, sequencing more than 6-fold deeper at 13 vs 2 million did not drastically increase gene detection (Fig. 2b). Differentially expressed genes detected by population RNA-seq were reliably detected by DRUG-seq at both read depths in ROC curve analysis (Supplementary Fig. 3b), and these genes separated samples into distinct treatment groups in response to compound treatment (Fig. 2c). Compound specific dose-dependent expression patterns in responses to treatment were maintained across platforms and read depths (Fig. 2d, Supplementary Fig. 3c as control). DRUG-seq is capable of capturing transcriptome changes related to specific perturbations even at lower read depths. This sequencing depth is comparable with previously published results^8^ and reduces sequencing cost to about 1/100^th^ of standard libraries ($3/sample on Illumina Hiseq 4000 platform). The continuing drop in sequencing cost will further reduce the cost of DRUG-seq.

### DRUG-seq can be used to profile compounds and cluster them based on their transcriptional profile

We utilized the DRUG-seq platform to profile a collection of 433 compounds with predominantly known targets (Supplementary Data 3) in triplicates for 8 doses (10 μM, 3.2 μM, 1 μM, 0.32 μM, 0.1 μM, 32 nM, 10 nM, and 3.2 nM) in the osteosarcoma U2OS cells. Optimum treatment time is compound and application dependent. We chose 12 h to balance detection of compound effectiveness and loss of material due to toxicity. Fifty-two out of the 433 compounds that induced transcriptome changes are also present in the Connectivity Map database (https://clue.io/). We compared the number of differentially expressed genes detected by DRUG-seq with the transcription impact score measured in Connectivity Map. In spite of different technology platforms, the two measurements correlate well (*r* = 0.80, Supplementary Fig. 3d), indicating compound potency differences are reliably captured in DRUG-seq.

**Fig. 3:**
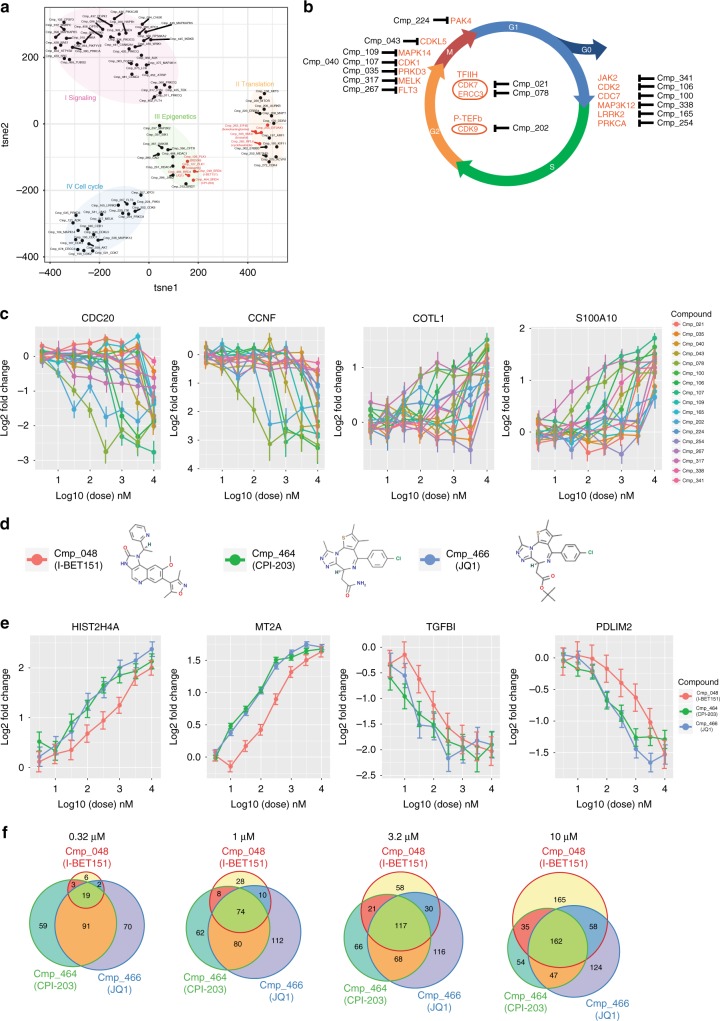
DRUG-seq profiling of compounds produces mechanistic insights. **a** tSNE clustering using 4289 dysregulated genes under compound treatment at 10 μM. Each compound and its target are labeled and closely clustered compounds with labeled common mechanisms are grouped by colors. **b** Compounds in cluster IV arranged based on the functions of their targets during cell cycle^21–33^. CDK7 and ERCC3, being part of TFIIH complex, are involved in DNA repair^34^. CDK9 is the catalytic subunit of transcription elongation complex P-TEFb^34^ **c** Dose-dependent gene expression changes under the treatment of compounds in **b**. Mean gene expression level and standard deviation (*n* = 3) for each compound and dose combination represented. **d** Structure of 3 compounds targeting Brd4. **e** Dose-dependent gene expression changes under the treatment of Brd4 compounds. Mean gene expression level and standard deviation (*n* = 3) for each compound and dose combination represented. **f** Venn diagram of the number of dysregulated genes under increasing dose by Brd4 compounds

Transcriptional signatures after compound treatment can group compounds with similar MoA and facilitate mechanistic studies of novel compounds^5^. Out of the 433 compounds, 88 were identified as potent compounds with more than 50 genes significantly changed (|log2(Fold Change)| > 1 and padj < 0.05). Using changes of these genes as measurements in tSNE clustering analysis, we observed distinct signatures (Fig. 3a). Known targets of compounds in the same cluster can functionally implicate common pathways affected under different treatments. MoA of compounds with unknown targets can be inferred from their neighbors in the cluster. For example, in cluster II, although Cmp_308 (brusatol) is a Small Molecule with Unknown Target(SMUT), it closely clusters with Cmp_263 (homoharringtonine), Cmp_253 and Cmp_282 (cycloheximide), which target *EIF4E*, *EIF2AK1* and *RPL6*, respectively, all components of the translation machinery. This suggests that Cmp_308 (brusatol) may also share similar MoA. Interestingly, this was supported by a recent report identifying inhibition of *Nrf2* by this compound through targeting the translation machinery^10^. While additional follow-up experiments will need to be conducted to confirm the newly proposed MoA, this is a valuable validation of our platform and in silico proof-of-concept.

Cluster IV consists of many compounds targeting cell cycle machineries, most of which govern G1-S or G2-M transition (Fig. 3a, b). Interestingly, many genes involved in cell cycle functions, such as *CDC20* and *CCNF*, were downregulated under these compound treatments (Fig. 3c), indicating systematic cell cycle perturbation by targeting a single component. However, dose-dependent kinetics of dysregulation of the same target is distinct for each compound, reflecting compound specificity and potency (Fig. 3c).

In cluster III, compounds targeting genes involved in epigenetic regulation, such as *Brd4, BrdT, HDAC1*, and *HDAC4*, are closely grouped (Fig. 3a). Cmp_126 (BI2536) and Cmp_127 (BI6727, volasertib), which target *PLK1*, are also nearby, indicating commonly affected pathways. Interestingly, recent publications classified both compounds as dual kinase-bromodomain inhibitors^11,12^. Of the three compounds targeting *Brd4*, Cmp_466 (JQ1) and Cmp_464 (CPI-203) are structurally related, while Cmp_048 (I-BET151) is more distinct (Fig. 3d). At 10 μM, there is significant overlap of genes affected by all three compounds, including genes involved in chromatin assembly/disassembly and cell death (Supplementary Fig. 5). Although all three compounds similarly impact gene expressions at high doses, Cmp_048 (I-BET151) has a much milder effect at mid to low doses while Cmp_466 (JQ1) and Cmp_464 (CPI-203) track each other closely (Fig. 3e). Few genes are dysregulated by Cmp_048 (I-BET151) at 0.32 μM even though it affects a similar number of genes at higher doses as the other two compounds (Fig. 3f). This not only indicates that DRUG-seq is a powerful tool to identify compounds with similar MoA, but also demonstrates that it is sensitive enough for detailed comparisons of related, yet distinct, compounds.

### DRUG-seq can be used to profile CRISPR knockout cells to uncover gene and compound function

**Fig. 4:**
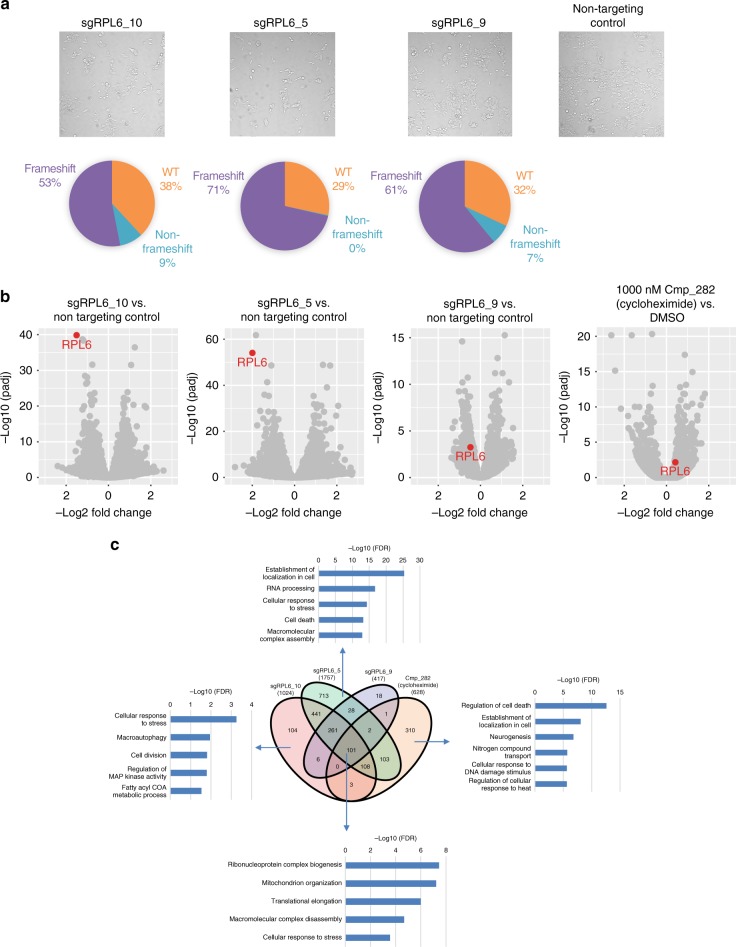
CRISPR KO results compared with compound treatment of the same target. **a** Upper: CRISPR knockout of RPL6 reduced confluency. Lower: Indel type breakdown of specific guide RNA treatment from average of 4 samples. **b** Differential gene expression analysis of RPL6 CRISPR knockouts and 1 μM Cmp_282 (cycloheximide) treatment targeting RPL6. Target gene RPL6 is highlighted in red. *n* = 4 for CRISPR samples and *n* = 3 for compound treatment samples. **c** Venn diagram of the number of differentially expressed genes in CRISPR knockouts and under 1 μM Cmp_282 (cycloheximide) treatment, and GSEA analysis of 101 core genes impacted in all CRISPR knockouts as well as Cmp_282 (cycloheximide) treatment, and CRISPR/compound treatment specific GSEA categories. Selected GSEA categories are each represented with –log10(FDR) value

Gene editing with CRISPR/CAS9 has increasingly become an important tool in early pharmacological target validation when target-specific compounds are not available. As proof-of-concept, we set out to compare the effect of CRISPR knockout and compound inhibition of a well-validated compound, Cmp_282 (cycloheximide), with its established target, *RPL6*, using DRUG-seq. The ability to multiplex many treatment conditions accommodates CRISPR control and treatment replicates all in the same plate, and the large number of genes profiled allows for comprehensive comparison. Two plates were set up for each experiment; one plate was processed through the DRUG-seq workflow, and a duplicate plate was processed and sequenced for specific CRISPR-induced indel generation to determine the efficiency of generation of loss of function alleles^13^. In addition, cell confluency was closely monitored to capture potential knockout phenotypes. CRISPR guides targeting *RPL6* demonstrated dramatic phenotypic changes. Four days post-transfection, markedly reduced confluency was observed in replicate wells and indel analysis confirmed significant frameshift mutations between 53 to 71% (Fig. 4a). In knockouts mediated by sgRPL6_10 and sgRPL6_5, *RPL6* level was among the most significantly reduced transcripts (65 and 75%, padj = 1.4 × 10^−40^ and 8.5 × 10^−55^, respectively), while knockout by sgRPL6_9 resulted in a much milder reduction (28%, padj = 0.0006) (Fig. 4b). Unlike CRISPR treatment, compound Cmp_282 (cycloheximide) for 12 h did not reduce *RPL6* mRNA (Fig. 4b). Interestingly, there is only a partial overlap between the transcriptomes of the CRISPR- and compound-treated cells, likely due to the different mechanism of CRISPR gene knockout vs. compound treatment and/or the difference in kinetics: Slow for CRISPR and rapid for compound treatment. Genes affected by compound treatment did partially overlap with genes dysregulated in CRISPR knockouts (Fig. 4c), many of which are involved in translation and rRNA metabolic processes. The result is expected for the function of a ribosomal subunit and a compound impacting the translational machinery. Using this set of genes, treated samples from both compound and CRISPR perturbations were clustered together and clearly separated from DMSO treatment and non-targeting control samples in hierarchical clustering (Supplementary Fig. 6), suggesting a shared mechanism under both treatments. Interestingly, there were many more genes that were uniquely affected by the compound and these include regulators of cell death, localization, and cellular response to DNA damage stimulus (Fig. 4c). This may indicate that Cmp_282 (cycloheximide) has additional unidentified targets.

### DRUG-seq has advantages over L1000 and RASL-seq

Besides significant cost reduction, another advantage of the DRUG-seq platform compared to L1000 and RASL-seq is the ability to directly measure > 10000 genes without computational inference, many of which were not included in L1000 assay (Supplementary Fig. 3a). To compare the accuracy of the two platforms, tSNE plus K-means clustering using genes either directly measured or measured + inferred in L1000 was compared with DRUG-seq results. Clustering in DRUG-seq was more accurate with better separations (Supplementary Fig. 7a-d). The 1351 differentially expressed genes detected by DRUG-seq but not by L1000 + inferred are enriched for pathways involved in mitochondria functions, tretinoin response, *EZH2* targets and genes located in 9q34 (Supplementary Data 4 and Supplementary Fig. 7e), underscoring the advantage of a more comprehensive transcriptome by DRUG-seq. This indicates that targeted approaches such as RASL-seq or L1000 capture some but not all transcriptional changes afforded by a less-biased approach such as DRUG-seq.

## Discussion

In summary, we developed a massively parallelized, automated, low-cost next-generation sequencing-based method to profile whole transcriptome changes under chemical and genetic perturbations and successfully applied it in an industrial high-throughput screening environment. DRUG-seq is a powerful tool that has advantages over other technologies such as RASL-seq, PLATE-seq and L1000. It is easier to perform, has higher throughput, is unbiased and is cost-effective. DRUG-seq is a powerful tool to assist novel compound mechanistic studies, compound repurposing efforts and identification of genetic transcriptional networks with CRISPR/CAS9-based gene knockout.

## Methods

### Cell lines and compound treatment

C2C12 (ATCC CRL-1772), 293 T (ATCC CRL-3216), and U2OS(ATCC HTB-96) cells were used in compound profiling experiments. Passage number was limited to 50 before being replaced with a new batch of cells. Cells were plated down to 50–80% density in 384-well plates or 24-well plates for at least 6 h before compound treatment. For 384-well treatment, compounds were added at half log decreasing dose for 8 doses: 10 μM, 3.2 μM, 1 μM, 0.32 μM, 0.1 μM, 32 nM, 10 nM, and 3.2 nM in triplicates with DMSO as a control. For population RNA-seq study, compounds were added to cells grown in 24-well plates at 10 μM, 1 nM, and 0.1 μM in triplicates with DMSO as a control. After 12 h, 384-well plates were collected and cells were lysed for DRUG-seq library preparation. Cells in 24-well plates were processed following the standard population RNA-seq procedure below.

### Population RNA-seq library construction

Cells were collected from wells and RNA was purified with RNeasy mini kit (Qiagen). RNA QC was carried out with Bioanalyzer RNA 6000 nano kit (Agilent). Population RNA-seq libraries were constructed according to manufacturer’s instruction (Illumina Truseq library construction), and sequenced on a Hiseq 4000 instrument with a minimum of 30 mil read depth.

### Population RNA-seq data analysis

Population RNA-seq data were processed using a standard RSEM workflow^14^. The resulting FPKM values were used for comparison with DRUG-seq. Gene detections were evaluated with NCBI Entrez genes present in Ensembl reference.

For clustering comparison between population RNA-seq and DRUG-seq, unsupervised meta-clustering was performed using the nclust1 R package available at (http://bcf.isb-sib.ch/nclust/v1/). Briefly, as similarity measures, Fisher Z-transformation of Pearson’s correlation is combined across datasets using random effect meta-analysis summary^15^. Average linkage is used as the clustering criteria^16^. For visualization of the dendrogram, the height of the tree branches corresponds to the correlation obtained by inversing the Fisher Z-transformation of the average linkage scores. Row clustering is performed on pooled data matrix. For visualization, they are stratified by the dataset of origin, but maintaining the ordering of the pooled data.

### DRUG-seq library construction and sequencing

Cells attached to wells were lysed directly in wells with 15 μl lysis buffer. Then plates were sealed and placed on a microplate shaker for 15 min at 900 rpm. Barcoded DRUG-seq RT primers (Supplementary Data 1) were dispensed into individual wells with an Echo liquid handler (Labcyte Inc), and cell lysate was transferred into 384-well PCR plates pre-dispensed in each well with RT mix and diluted ERCC mix1 (Thermo Fisher). Plates were incubated at 42 °C for 2 h. Material from each well in a 384-well plate was pooled into a single sample, purified with DNA clean & concentrator-100 kit (Zymo Research) and Agencourt RNAClean XP beads (Beckman Coulter). After ExoI treatment, material was pre-amped with DRUG-seq PCR primers and purified. The pre-amped material was fragmented with Nextera enzyme (Illumina), and individual libraries were indexed and quantitated with qPCR before sequencing on a Hiseq 4000 (Illumina). See Supplementary Information for specific barcode and primer sequences and detailed protocol.

### DRUG-seq data analysis

DRUG-seq data processing was carried out with a custom script. Briefly, 10-base well position barcodes and 10-base UMI information from read1 fastq are first incorporated into read2 fastq as part of sequence ID. Then read2 sequence was aligned to transcriptome by STAR^17^ with hg38 reference index and Ensembl gene references. The resulting uniquely mapped alignments are then demultiplexed and the transcript UMI for each gene is counted for each sample well. A gene expression matrix is then used for differential gene expression analysis with DESeq2 in R^18^ for each triplicate samples representing unique compound/dose combination with DMSO treatment as control.

Clustering analysis was carried out using tSNE method^19^. Compounds were selected if at 10 μM more than 50 genes in differential gene expression analysis meet the cutoff criteria (padj < 0.05, |log2(Fold Change)| > 1). Then log2(Fold Change) compared to DMSO was quantile normalized. For each qualified compound, up to 200 differentially expressed genes were selected as candidate genes to avoid a dominating effect by potent compounds. Then candidate gene lists for all compounds were combined, resulting in 4289 genes total, and the quantile normalized log2 (Fold Change) for each gene under each compound treatment was used as the measurement. Up to 3000 iterations were performed to generate optimum clustering using the tsne package in R. Over-represented gene categories were generated using gene set enrichment analysis^20^.

Gene selection for hierarchical clustering analysis of Cmp_078 (triptolide) and Cmp_263 (homoharringtonine) was carried out similarly, with up to 1000 candidate genes selected for each compound. The quantile normalized log2 (Fold Change) was used to calculate Euclidean distance in clustering analysis.

From population RNA-seq results, differentially expressed genes with log2 (Fold Change) > 2 were used as true positives in ROC curve analysis for DRUG-seq samples with the same compound/dose combination.

### DRUG-seq profiling of CRISPR mutants

To profile CRISPR mutations, synthetic targeting and control gRNAs were reverse transfected using Lipofectamine RNAiMAX (Thermo Fisher) in 4 replicates to a U2OS cell line stably expressing Cas9 at 2500 cells per well in a 384-well plate. Cells were monitored daily after transfection and collected at day 4 for indel and transcriptome profiling. Library construction, sequencing and data analysis were carried out the same way as compound-treated samples. Differentially expressed genes were selected with cutoff of padj < 0.05 and |log2(Fold Change)| > 0.6 for both CRISPR mutations and Cmp_282 (cycloheximide) treatment. Over-represented gene categories were generated using gene set enrichment analysis^20^.

### CRISPR indel detection

CRISPR indel detection were carried out as previously described^13^. To prepare the samples for sequencing, two rounds of PCR were performed. The first round of PCR utilized locus-specific primers to amplify the edited region (Supplementary Data 2). The product formed during the first round was then used as a template for a second round of PCR to add dual indices compatible with the Illumina system. Libraries were quantitated by qRT-PCR and subsequently sequenced on the Illumina MiSeq system. For sequence analysis, raw reads were aligned to a reference sequence, then tallied based on genotype. Finally, tallied genotypes were binned into one of three categories: wild type, in-frame, and frameshift.

### Statistics

Statistical test used in differential gene expression analysis is provided by DESeq2 R package^18^. All sample correlations are calculated using Pearson method with two tailed *t* test. GSEA employs statistical test detailed in ref. ^20^. All barplots are overlaid with scatter plots to show individual data points with the exception of Fig. 2b, where it was not possible to plot all the points and the GSEA analysis in Fig. 4c, where each bar represents a single -log10(FDR) value.

### Computer code availability

All custom computer codes are available upon reasonable request from the corresponding author (A.K.).

### Note on cell lines

C2C12, 293 T, and U2OS are not on the latest list of ICLAC commonly misidentified cell lines, and the identity of a particular cell line is immaterial to the technical assessment of DRUG-seq. Cell lines are also obtained from ATCC, which authenticates cell lines by STR testing. Internally, all cells are routinely tested for mycoplasma contamination.

## Electronic supplementary material

Supplementary Information

Supplementary Data 1

Supplementary Data 2

Supplementary Data 3

Supplementary Data 4

Peer Review File

Description of Additional Supplementary Files

## Data Availability

The RNA-seq data has been deposited at GEO under the accession code GSE120222. All other data are available upon reasonable request from the corresponding author (A.K.).
