# Supplementary material for: DRUG-seq for miniaturized high-throughput transcriptome profiling in drug discovery
Source: Nat Commun. 2018 Oct 17;9:4307. doi: 10.1038/s41467-018-06500-x (PMC6192987; doi:10.1038/s41467-018-06500-x)
Supplement: Supplementary file 1 — Supplementary Information [file 41467_2018_6500_MOESM1_ESM.pdf]

DRUG-seq: Miniaturized High Throughput Transcriptome Profiling for Drug Discovery
Ye et al.

This file contains:

1. Supplementary Methods
2. Supplementary Figure 1: Comparison between PLATE-seq and DRUG-seq
3. Supplementary Figure 2: Mixed species experiment to assess cross well contamination
4. Supplementary Figure 3: DRUG-seq compared with population RNA-seq and L1000
5. Supplementary Figure 5: GSEA of genes affected by Brd4 compounds at 10uM
6. Supplementary Figure 6: Compound and genetic perturbation samples clearly separate from controls
7. Supplementary Figure 7: Using L1000 genes reduces tSNE clustering separation

Supplementary method:
Lysis buffer: 50mM Tris-HCl pH8.0 (Life Tech 15568-025), 75mM KCl (Ambion AM9640G), 6% Ficoll PM-400 (Sigma F5415-25ML), 0.15% TritonX-100 (Sigma T8787-100ML). Add RNaseOUT (Thermo Fisher) to 0.5U/ul before use.
RT mix: 1X RT buffer (from Thermo Fisher Maxima H Minus Reverse Transcriptase EP0753), 8mM MgCl (AM9530G), 0.8uM strand switching primer (TSO), 0.08uM dNTP, 0.3U/ul RNaseOUT (Thermo Fisher), 8U/ul Maxima reverse transcriptase.

Prepare RT mix and dispense 2.5ul into each well in a 384 well PCR plate. Then add 100nL 1:100 diluted ERCC mix1 (Thermo Fisher) per well using Echo liquid handler (Labcyte Inc.). Keep on ice.
After 12 hours of compound treatment, aspirate off media and wash cells once with PBS. Add 15ul lysis buffer to each well. Seal plates and place on a microplate shaker for 15 minutes at 900 rpm. Observe under microscope to ensure sufficient lysis.
Use Echo liquid handler to dispense 10nl 1uM barcoded DRUG-seq RT primers for each well.
Transfer 15ul cell lysate into RT mix plate. Seal and incubate at 42C for 2 hours.
Pool samples from 384 well plate into a single sample. Purify with DNA clean & concentrator-100 kit (Zymo Research). Elute in 150ul H2O.
Mix 150ul Agencourt RNA clean beads (Beckman Coulter) with materials eluted from columns above. Allow binding for 5min. Then use magnet to pellet beads. Wash twice with freshly made 80% EtOH. Allow to dry completely and elute in 32ul H2O.
Add 4ul ExoI buffer and 4ul ExoI (NEB M0239S) to the elute, incubate at 37C for 30 minutes then heat inactivate at 85C for 15 minutes. Hold at 4C
Add 50ul 2XKapa HIFI PCR ReadyMix (Kapa Biosystems, KK2602), 10ul 10uM DRUG-seq PCR primer and amplify material using following program: Preheat thermal cycler to 96C, then incubate at 96C for 1 min, 5 cycles of:98C 20s, 58C 4min, 72C 6min; 13 cycles of: 98C 20s, 60C 30s, 72C 6min; 72C 10min and hold at 4C.

Mix 100ul Agencourt RNA clean beads with PCR mix (1:1 beads ratio). Allow binding for 5min. Then use magnet to pellet beads. Wash twice with freshly made 80% EtOH. Let beads dry and elute in 11ul H2O. Run 1ul on Bioanalyzer DNA high sensitivity chip (Agilent).
Mix 10ul pre-amp material with 10ul nuclease-free water, 25ul TD buffer and 5ul TDE1 buffer (Nextera kit from Illumina), incubate for 5min at 55C and hold at 10C.
Purify with Qiagen MinElute PCR Purification Kit. Elute in 25ul nuclease-free water.
Mix with 15ul NPM, 5ul DRUG-seq_p5_PCR primer (5uM) and 5ul DRUG-seq indexing primer mix (comprised of 4 indices for each library, 5uM). Run following PCR reaction: 72C for 3min, 95C for 30sec, 15 cycles of: 95C for 10sec, 55C for 30sec, and 72C for 30sec; 72C for 5min and hold at 4C.
Mix 50ul AMPure beads with PCR mix (1:1 beads ratio). Allow binding for 5min. Then use magnet to pellet beads. Wash twice with freshly made 80% EtOH. Let beads dry and elute in 20ul H2O.
Size select between 200bp to 600bp fragment size with Pippin 2% gel cassette (Sage Science). Run 1ul on Bioanalyzer DNA high sensitivity chip.
Quantitate library with qPCR and follow manufacturer's instruction for sequencing using custom read1 primer.

	Avg # of genes detected	# of cells needed per well	format	RNA purification needed	processing time	library construction cost per sample
PLATE-seq	10200	10000	96	yes	3 hrs RNA purification before RT	$15
DRUG-seq	11000	2500	384, 1536	no	direct lysis and RT	$0.9

Supplementary Figure 1: Comparison between PLATE-seq and DRUG-seq

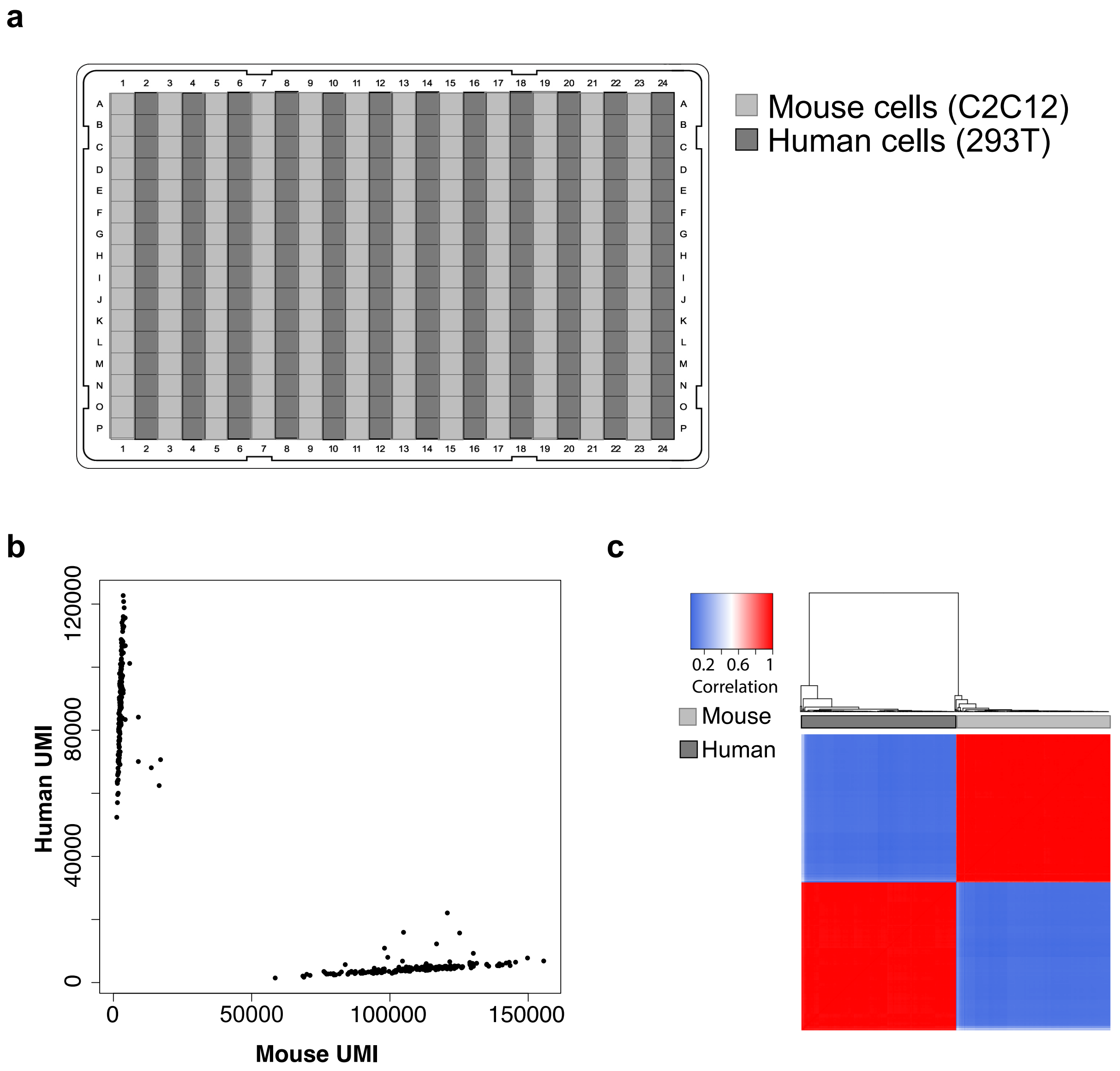

Supplementary Figure 2: Mixed species experiment to assess cross well contamination. (a) Mouse and human cells are plated in interweaved pattern and processed according to DRUG-seq workflow. **(b)** After reads mapping to combined mouse and human reference genome, UMI in each well was tallied for both species and plotted. More than 98% of wells have dominantly species specific UMI with purity > 96%. Purity = species specific UMI/total UMI. **(c)** Sample Pearson correlations are high among wells with cells from the same species, but low among wells with cells from different species.

a

Platform	Average L1000 genes detected	Average additional entrez genes detected
Population RNA-seq	100%	16084
DRUG-seq 13mil/well	90%	11200
DRUG-seq 2mil/well	83%	9750

b

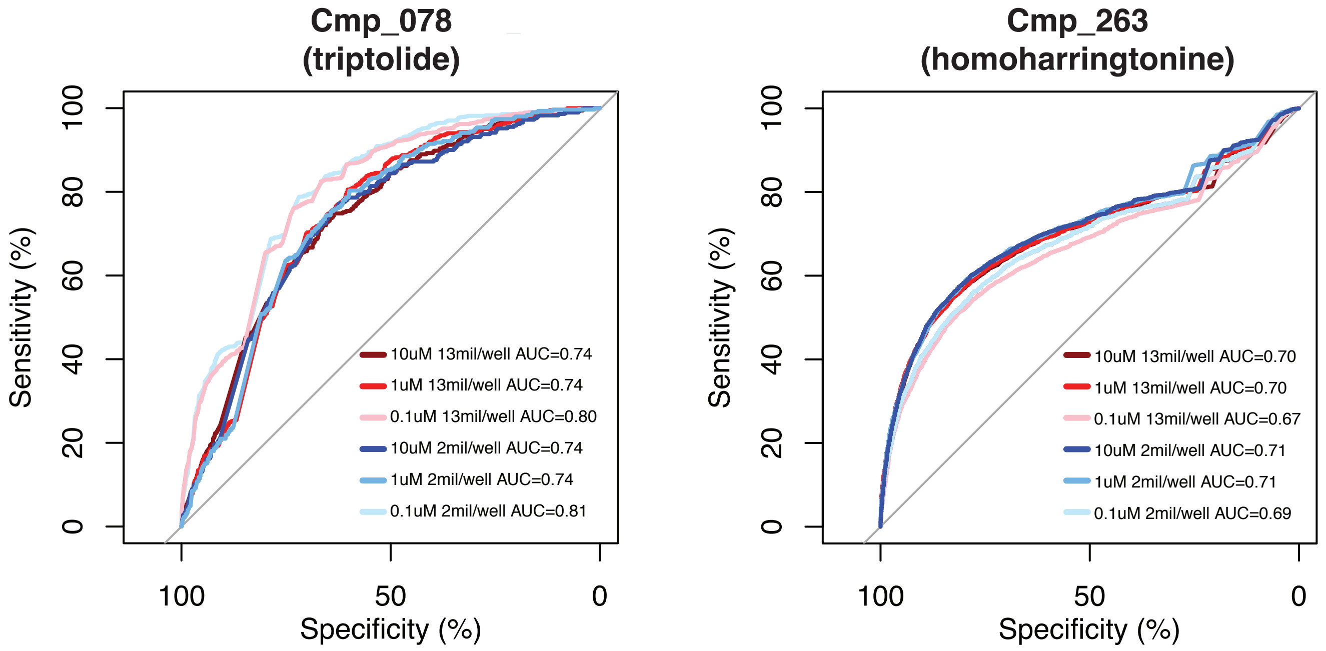

c

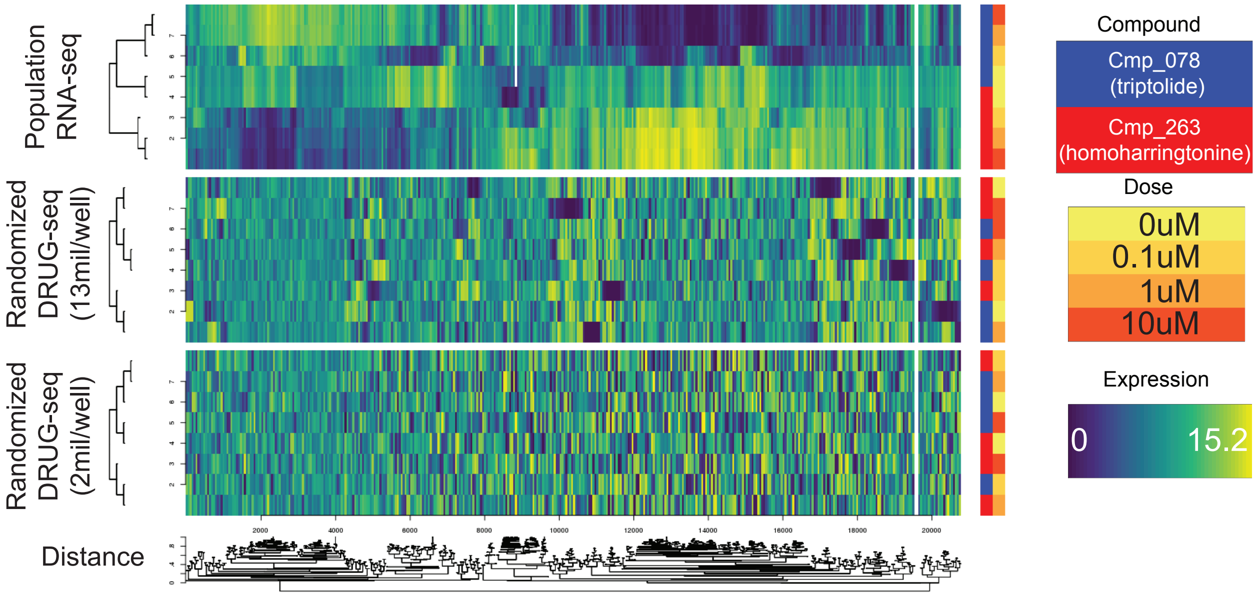

d

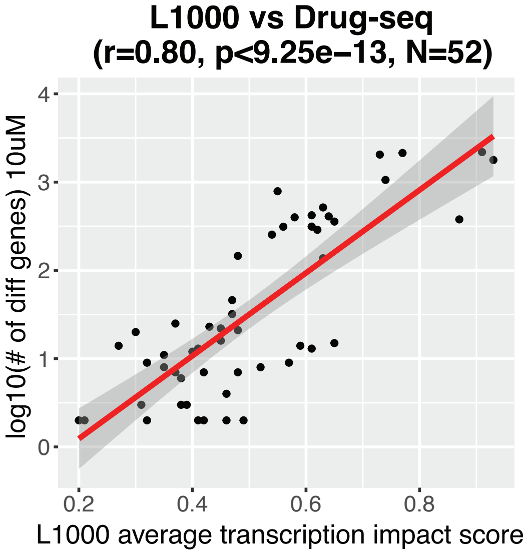

Supplementary Figure 3: DRUG-seq compared with population RNA-seq and L1000. (a) Detection of L1000 landmark genes were assessed in population RNA-seq and DRUG-seq libraries sequenced at different read depth. Number of additionally detected entrez genes were also noted. **(b)** ROC curve analysis for genes measured by DRUG-seq in samples treated with Cmp_078 and Cmp_263 at multiple doses. Genes with log2(Fold Change) > 2 from population RNA-seq were selected as true positives. **(c)** Control analysis for Figure 2c. Same clustering analysis as Figure 2c were carried out but with randomized DRUG-seq samples. **(d)** Transcription impact scores in L1000 was compared with the number of differentially expressed genes in DRUG-seq platform using compounds demonstrating differential gene expression in DRUG-seq which are also present in L1000 database (N=52, correlation coefficient based on Pearson correlation).

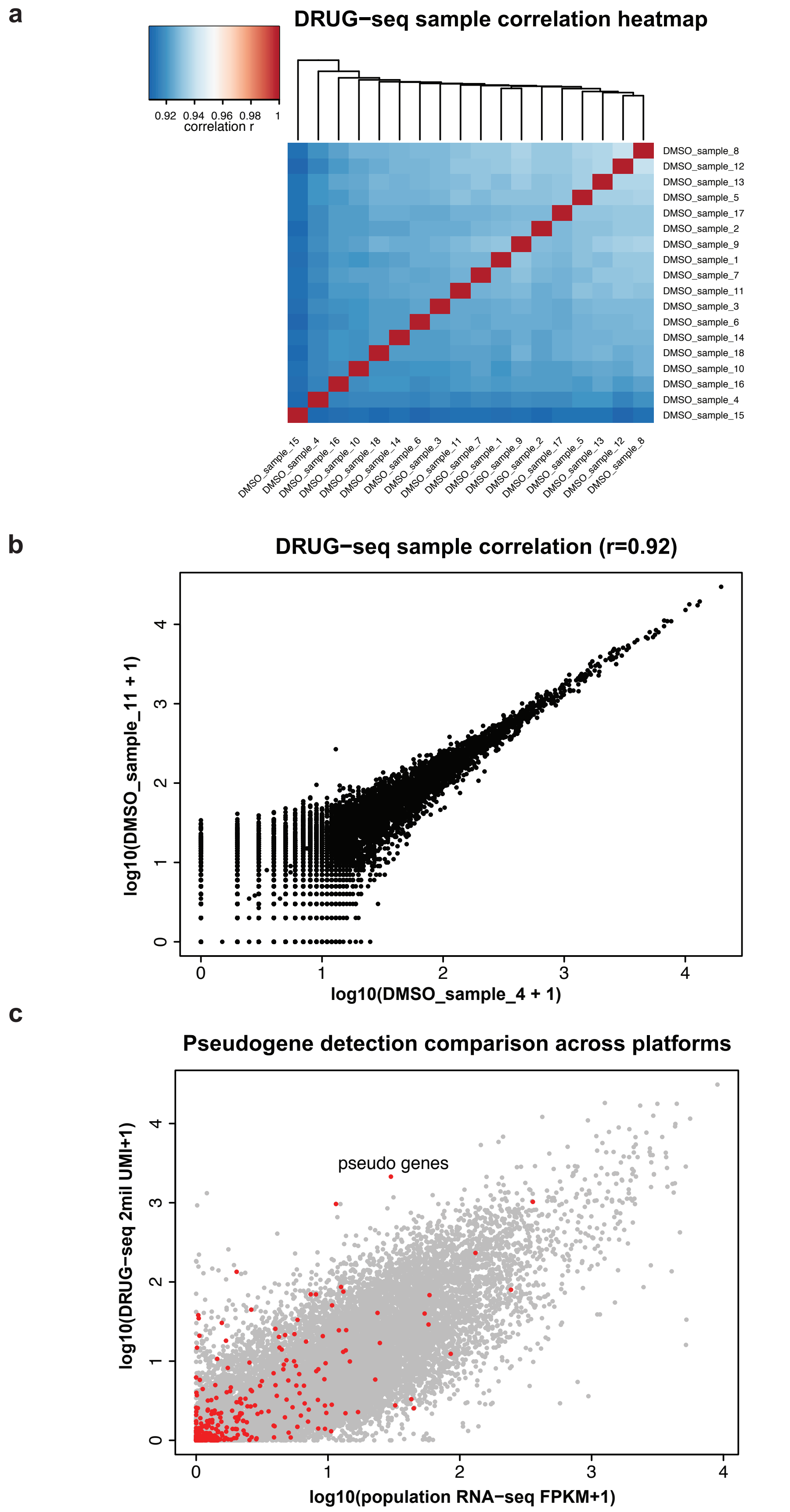

Supplementary Figure 4: Highly consistent detection of genes in DRUG-seq library sequenced at 2mil/well. (a) Gene expression are highly correlated among 18 samples using Pearson correlation coefficient. **(b)** Gene expression pattern are highly correlated in randomly selected 2 samples. **(c)** 3’ based RT reaction used in DRUG-seq does not bias detection of pseudo genes.

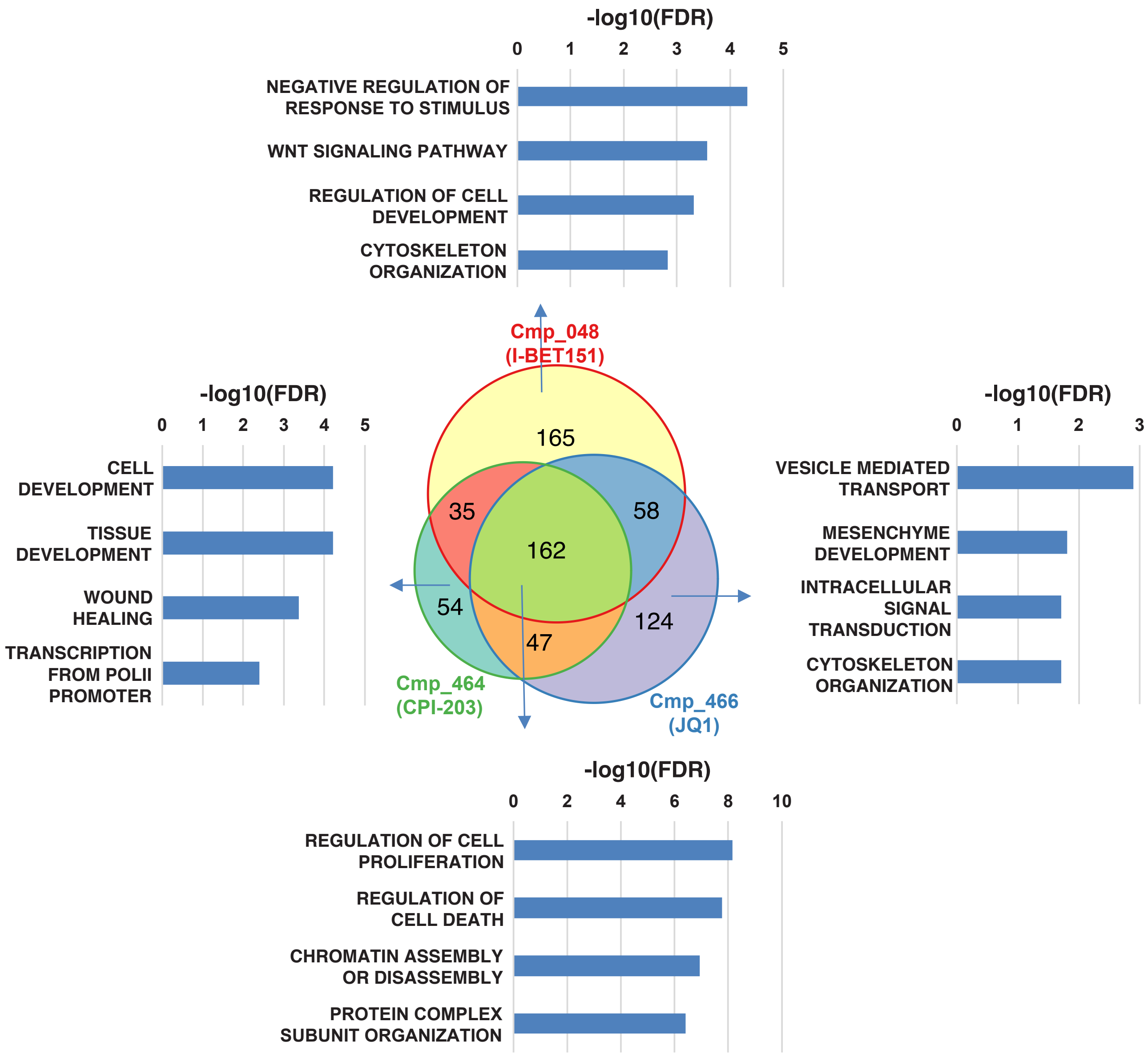

Supplementary Figure 5: GSEA of genes affected by Brd4 compounds at 10uM. The numbers of overlapping and unique genes for compounds targeting Brd4 are illustrated. GSEA was carried out using genes affected by all 3 compounds, and for genes uniquely affected by each compound. Only selected categories with their FDR values are listed.

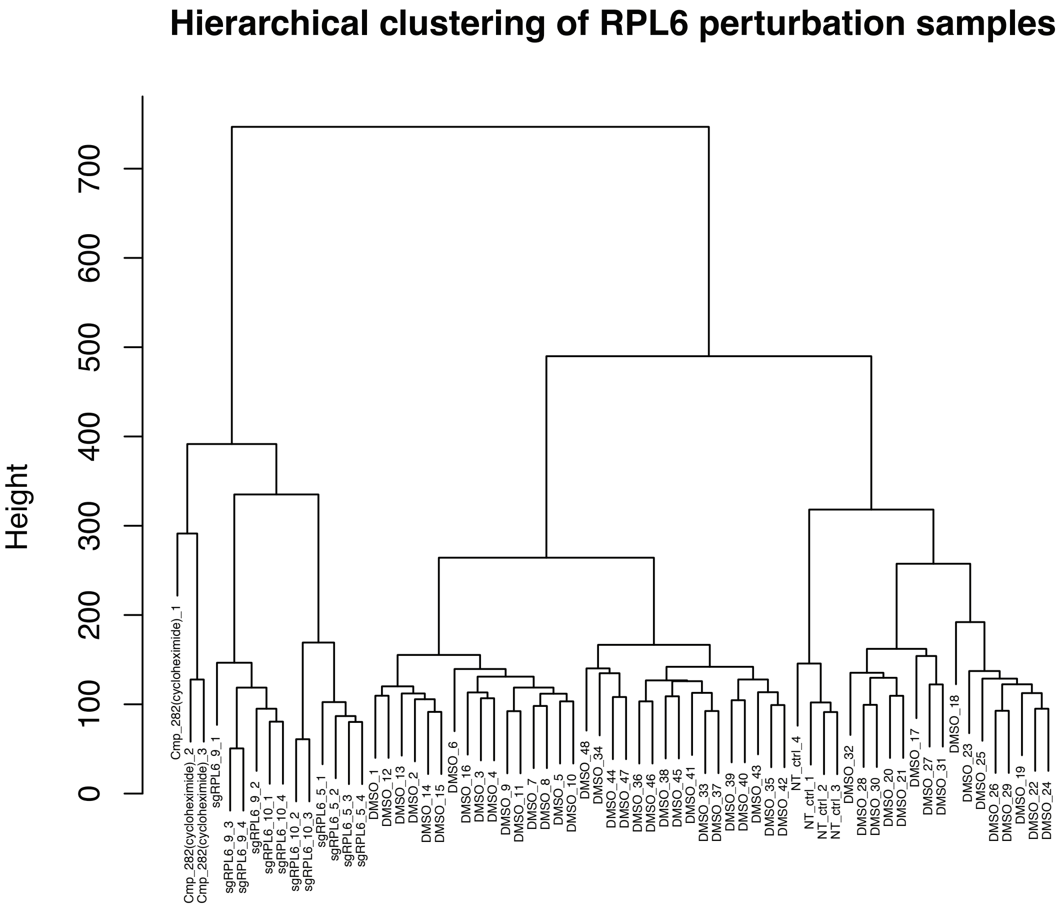

Supplementary Figure 6: Compound and genetic perturbation samples clearly separate from controls. Compound or CRISPR treated samples are clustered together in hierarchical clustering using common differentially expressed genes, while DMSO and non-targeting guide controls are clustered together.

a

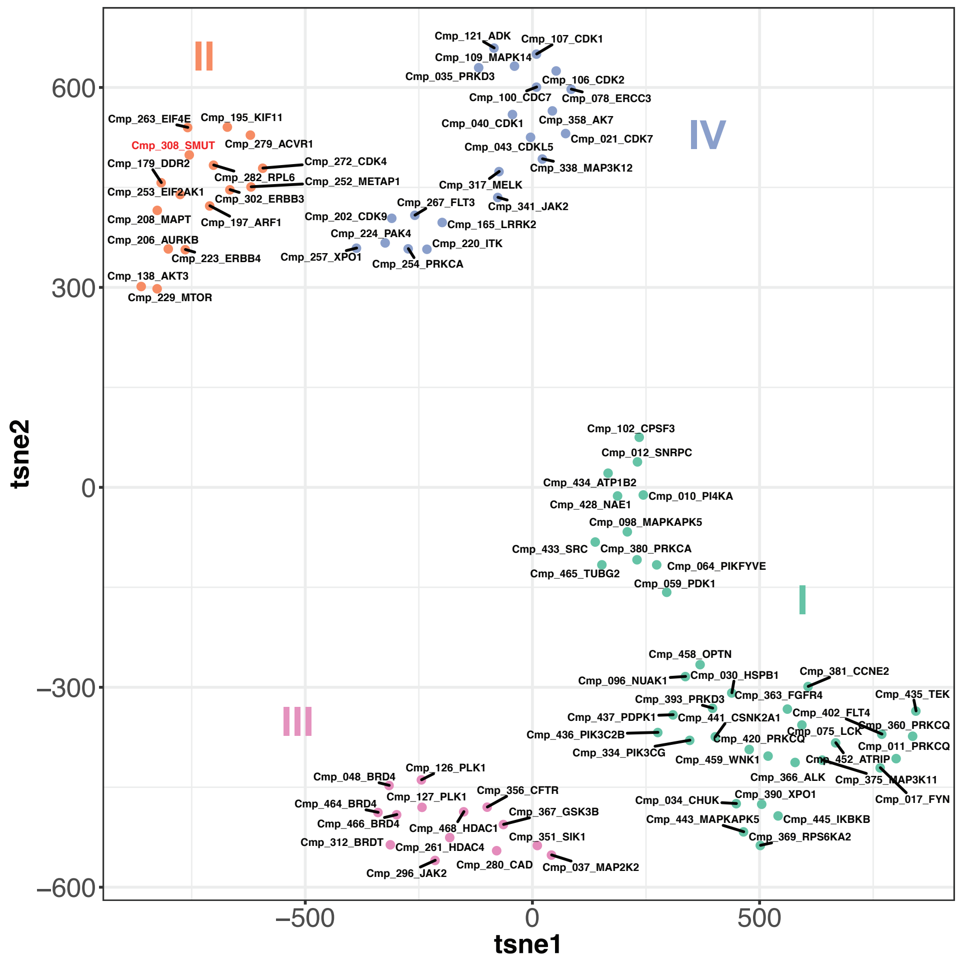

II
IV
I
III
tsne1
tsne2
Cmp_308_SMUT

b

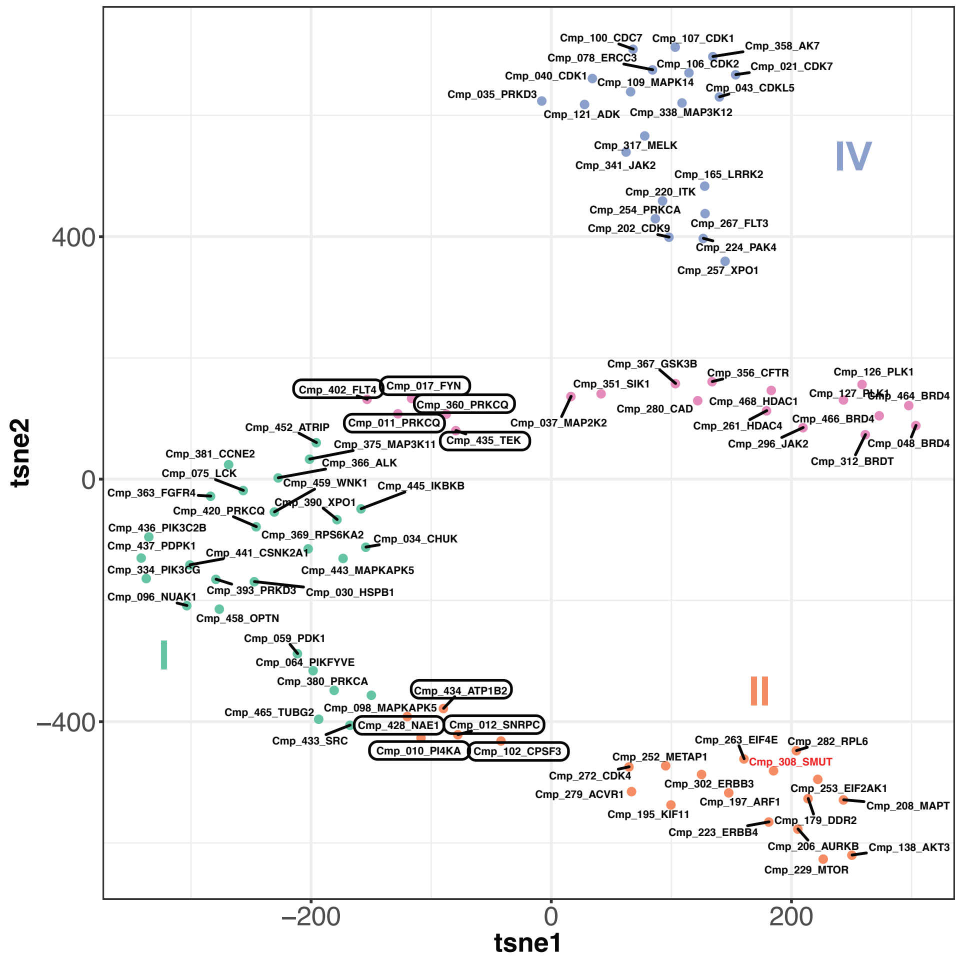

IV
I
II
tsne1
tsne2
Cmp_308_SMUT

c

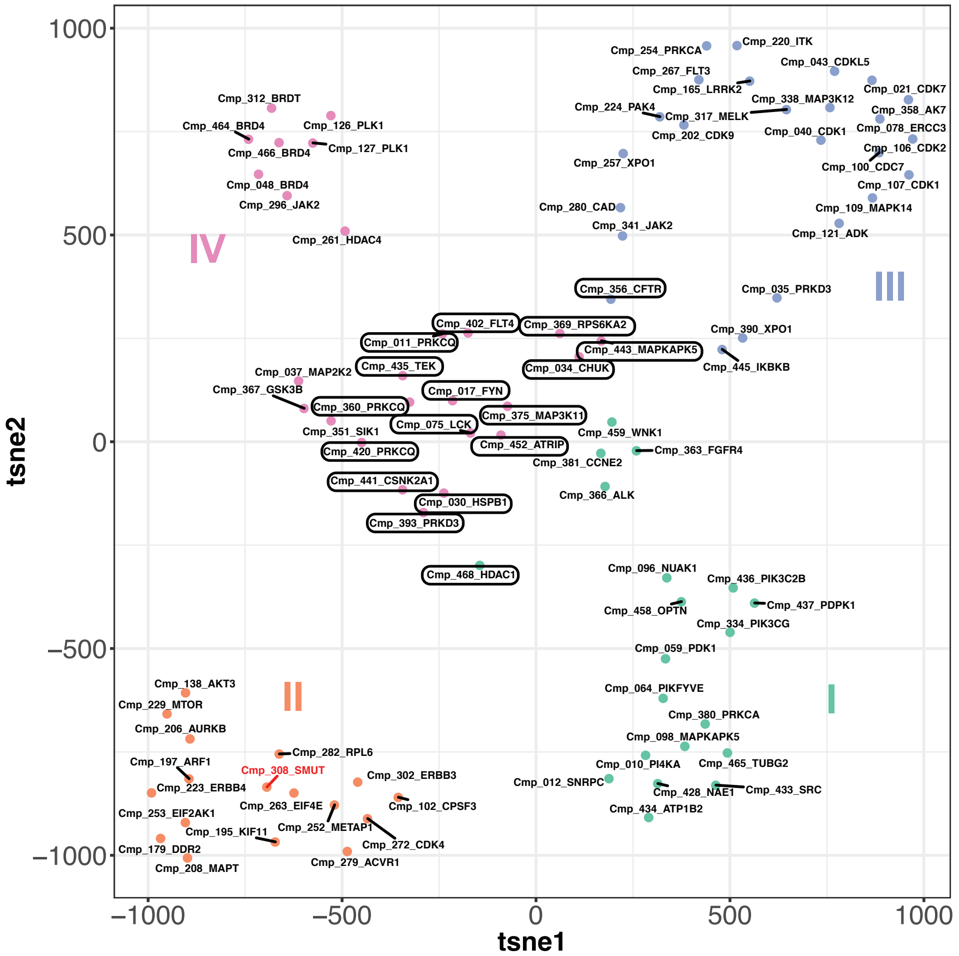

d

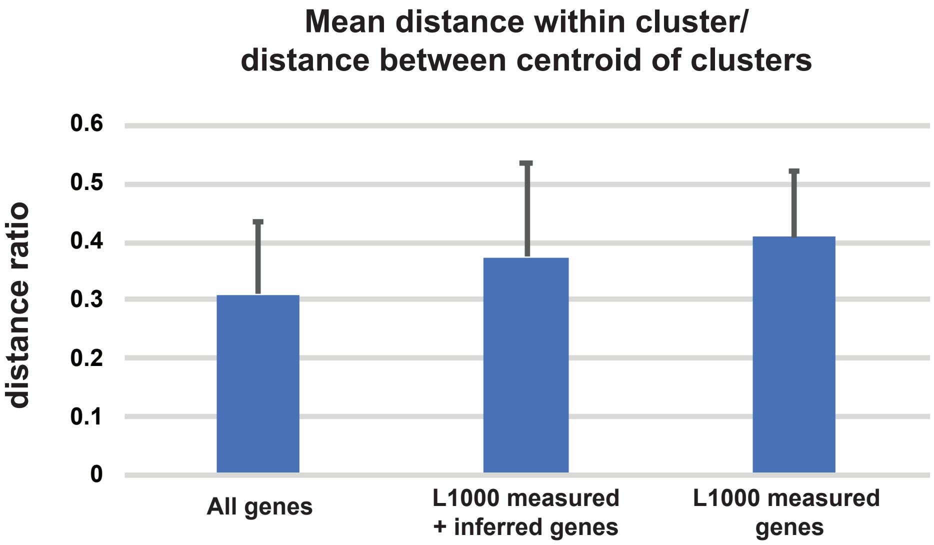

e

	# Genes in Gene Set (K)	# Genes in Overlap (k)	p-value	FDR q-value
GO_MITOCHONDRION	1633	91	2.86E-23	5.08E-19
GO_ENVELOPE	1090	67	1.65E-19	9.79E-16
MARTENS_TRETINOIN_RESPONSE_DN	841	57	1.16E-18	4.14E-15
GGGCGGR_SP1_Q6	2940	117	6.63E-18	1.96E-14
LU_EZH2_TARGETS_UP	295	33	1.59E-17	4.04E-14
GRAESSMANN_APOPTOSIS_BY_DOXORUBICIN_UP	1142	64	1.01E-16	2.00E-13
chr9q34	267	30	4.15E-16	7.38E-13

Supplementary Figure 7: Using L1000 genes reduces tSNE clustering separation. (a) tSNE clustering plus k-means clustering with the same set of 4289 genes used in Figure 3. Compounds belonging to the same K-means clusters are colored the same. **(b)** Same clustering analysis as in (a) but with genes overlapped with L1000 measured and inferred gene list (2938 genes total). 10 circled compounds are the ones mis-categorized to the wrong clusters compared to (a). **(c)** Same as above analysis but with genes overlapped with L1000 measured gene list only (394 genes total). 17 circled compounds are mis-categorized. **(d)** The ratio of mean intra cluster distance to inter cluster distance as measurement of cluster separation. Using L1000 genes only decreases cluster separation (N=88). (e) Gene set enrichment analysis (GSEA) for genes detected in DRUG-seq but not in L1000.
