# Supplementary material for: DRUG-seq for miniaturized high-throughput transcriptome profiling in drug discovery
Source: Nat Commun. 2018 Oct 17;9:4307. doi: 10.1038/s41467-018-06500-x (PMC6192987; doi:10.1038/s41467-018-06500-x)
Supplement: Supplementary file 7 — Description of Additional Supplementary Files [file 41467_2018_6500_MOESM7_ESM.docx]

**Title:** Supplementary Table I

**Description:** Barcoded DRUG-seq RT primers

**Title:** Supplementary Table II

**Description:** Additional primers used in this study

**Title:** Supplementary Table III

**Description:** List of compounds profiled in this study and their potential targets

**Title:** Supplementary Table IV

**Description:** Differentially expressed genes under compound treatment detected by DRUG-seq but not in L1000 measured or inferred gene lists” from the table of contents (these are the titles for the Description of Additional Supplementary Materials file that will need to be generated)
