# Supplementary material for: DRUG-seq for miniaturized high-throughput transcriptome profiling in drug discovery
Source: Nat Commun. 2018 Oct 17;9:4307. doi: 10.1038/s41467-018-06500-x (PMC6192987; doi:10.1038/s41467-018-06500-x)
Supplement: Supplementary file 2 — Supplementary Data 1 [file 41467_2018_6500_MOESM2_ESM.pdf]

Reviewers' Comments:

Reviewer #1:
Remarks to the Author:
In this manuscript, Ye et al develop a cheap and high-throughput method for profiling transcriptomes in well plates. This method is then used to study a number of drug compounds to determine their mechanism of action. The work presented is potentially interesting, though it is a technology that is quite similar to the previously described PLATE-Seq, and therefore would benefit from more direct comparison of this technology with related techniques. In addition, there are a number of major and minor comments below which should be addressed.

Major comments:
1. A key issue in the paper is a lack of direct demonstration that shows how perturbation of a benchmarking compound compares with a discovery (unknown) compound that is then subsequently validated by target ID or CRISPR.
2. There is an overall lack of clarity in describing compounds, their targets, labeling, etc (see below).
3. PLATE-Seq seems to be a comparable system, and a more thorough comparison of the technologies in terms of capabilities would be in order. A table outlining the different characteristics for the technologies described (e.g. # of transcripts detected, # of cells needed, cost, work) may be helpful to highlight the advantages.
4. An interesting comparison to make with the L1000 system would be to perform similar clustering. It would be interesting to do the clustering for the compounds using only the L1000 genes to demonstrate the strength of the system and the amount of power that is gained by sequencing more than the landmark transcripts.
5. For Figure 4, it may be worth expanding on the utility of the compound vs. genetic perturbation. The RPL6 mRNA not dropping during compound treatment isn't surprising, so it was not clear what the takeaway is from the comparison. Are the additional areas considered off-target (i.e. side effects) or at least non-RPL6 mediated effects? I think clarification here could be helpful.
6. In Figure 4, how is it possible to compare cyclohex rna seq pattern w/ rpl6 pattern based on volcano plots? It would be better to have PCA analysis or something else here; clustering would be best.
7. For the CRISPR experiment, how long were the cells given to generate indels? Was the Cmp_282 treatment comparison in Figure 4 for the same amount of time or 12 hours as in the compound characterization? It is probably hard to synchronize the drug with the editing event, but if the times are different, it could be acknowledged as potential difference between the treatments. For example, it could be that the extra effects picked up by DRUG-Seq are acute but the CRISPR cells have adapted during the extended recovery period to not show those transcriptional effects.

Minor comments:
1. line122/123 (and throughout the manuscript really): it will be clearer in most cases to refer to compounds by name (here, brusatol)
2. line 125-6: "Together our result suggest that Nrf2 translation is Cap-dependent and requires EIF4E function and further defines the MOA of Cmp_308" is quite a stretch without more discussion of previous paper cited. It would be better to moderate claim and just suggest the association if there is not to be direct follow-up
3. line 161: for clarity, just refer to this compound as cycloheximide
4. Line 9; phrasing in abstract to 'screen all targets at once' is confusing. really this is measurement of RNAs that are perturbed rather than the target of the drug. writing clarity could be improved with a transition sentence in abstract describing the general notion that rnaseq can be used as a proxy for drug effect
5. the authors claim of ability to detect nuanced perturbation differences from related drug molecules is not supported
6. choice of unknown molecules to begin with is odd and makes benchmarking difficult, e.g. in line 83, compounds (Cmp_078 and Cmp_263). what are these? where did they come from? why not

start with e.g. taxol or something? in any case, some kind of description/justification of these choices is needed
7. The authors then proceed in Fig. 3 directly to a tsne plot, not really discussing which compounds are used for benchmarking...are these the known targets or the predictions from the analysis here?
8. In fig. 2d, need to label all axes, dendograms directly on panel

Reviewer #2:
Remarks to the Author:
Here Ye et al describe the development of a novel method, DRUG-Seq, that allows for a low cost high-throughput automated platform for the generation of cellular transcriptome data. In particular, the authors apply this method to compound screening and demonstrate that with relatively low levels of sequencing, they are able to capture a significant portion of the transcriptome that can be used to cluster compounds with similar mechanisms of action. Establishing cost-effective high-throughput methodologies for screening (whether it be libraries of compounds, genes or gene variants) is useful to a broad scientific community. While the experiments described generally support the authors claims, additional data clarifying the performance of DRUG-Seq versus other more and less comprehensive methods (i.e. RNA-seq and L1000 candidate gene analysis) should be addressed.

1. Figure 2b shows that RNA-seq is able to detect a larger number of genes with FPKM/UMI distribution 0-1 and 10-100, but unexpectedly there is not better detection of the other gene subgroups. Please explain. In particular, why would RNA-seq detect a smaller number of genes in the >100 category compared to 13mil/well DRUG-seq?
2. How consistent are the results (in terms of what genes are detected and their quantified expression levels) for DRUG-seq libraries sequenced at 2 million reads/well?
3. Only the 3' end of the transcripts are sequenced. Presumably this is a limitation due to the barcoding, but also this reduces the need for extensive sequencing. Some discussion should be addressed to consider the caveats of this approach. For example, for genes with similar 3' ends (such as pseudogenes, etc.), how does the limited sequencing impact alignment?
4. The use of DRUG-seq for the evaluation of CRISPR knock-outs is a reasonable idea, but the manuscript flow needs improvement. What is the motivation for testing RPL6 knockout? This sentence, "Unlike CRISPR treatment compound Cmp_282 didn't reduce RPL6 mRNA". What is the relevance of Cmp_282 in this section? Is it an inhibitor of RPL6?
5. Since the 2mil/well DRUG-seq was able to capture almost the same information at L1000, it would be of interest to use the DRUG-seq data to approximate the dataset that would be collected by L1000, and then perform the clustering (Figure 3) to show whether DRUG-seq does provide superior clustering compared to methods that detect smaller numbers of transcripts.
6. The information in the supplementary tables is cut off.

Point-by-point rebuttal for Reviewer 1:

Reviewer 1

1. A key issue in the paper is a lack of direct demonstration that shows how perturbation of a benchmarking compound compares with a discovery (unknown) compound that is then subsequently validated by target ID or CRISPR.

We agree with the reviewer and think we over stated our observation a bit and have modified the text (lines 123 to 137) appropriately to highlight the value of DRUG-seq. The focus of the paper is on the cost-saving and high throughput value of DRUG-seq platform. The examples showcased in **Figure 3** and **Figure 4** cover a wide range of mechanisms including cell cycle disruption, epigenetics, transcription and translation, and the consistent clustering pattern strongly support the underlying mechanisms. In addition, at the time of carrying out the experiment, we did independently identify previous unknown mechanism of a compound Cmp_308 (brusatol) from our analysis (**Figure 3a, lines 130 to 137**), and the finding was further supported by another publication (reference 10). We are interested in following up on novel discoveries but feel that the intensive validation required to confirm a novel discovery is outside of the scope of this paper. Studies like this warrant a second publication and the work in this manuscript is intended to be a proof-of-concept to demonstrate our method.

2. There is an overall lack of clarity in describing compounds, their targets, labeling, etc. (see below).

We thank the reviewer for pointing this out. Not all compounds have conventional names, and most of those names are lengthy and complex. To systematically track compounds and facilitate automation, we organized compounds with unified naming scheme. All compound naming, formulas and their targets are compiled in **Supplementary Table III**. We also followed reviewer's suggestion and modified the main text to include more recognizable compound synonyms when available to facilitate reading.

3. PLATE-Seq seems to be a comparable system, and a more thorough comparison of the technologies in terms of capabilities would be in order. A table outlining the different characteristics for the technologies described (e.g. # of transcripts detected, # of cells needed, cost, work) may be helpful to highlight the advantages.

We agree with the reviewer. PLATE-seq and DRUG-seq are similar and detect a similar number of transcripts. However, DRUG-seq has many advantages. It is 1/15th the cost, doesn't need a lengthy RNA purification step and is amenable to high throughput screening in 384 or 1536 well plates. A table comparing the two methods is attached here and added as **Supplementary Figure I**:

	Avg # of genes detected	# of cells needed per well	format	RNA purification needed	processing time	library construction cost per sample
PLATE-seq	10200	10000	96	yes	3 hrs RNA purification before RT	$15
DRUG-seq	11000	2500	384, 1536	no	direct lysis and RT	$0.9

4. An interesting comparison to make with the L1000 system would be to perform similar clustering. It would be interesting to do the clustering for the compounds using only the L1000 genes to demonstrate the strength of the system and the amount of power that is gained by sequencing more than the landmark transcripts.

L1000 assay measures only about 1000 genes, with computational algorithms to infer the expression of additional 10000 genes. In DRUG-seq, all gene expression levels were measured directly. When clustering DRUG-seq data, we selected up to 200 of differentially expressed genes ($p<0.05$ and $|\log2(\text{Fold Change})| >1$) for each compound to avoid dominating effect by compounds with many dis-regulated genes (see **Methods**), resulting in 4289 genes included in the tSNE clustering in **Figure 3a**. Of these, 394 are directly measured in L1000 platform, and 2938 are either measured or inferred in L1000 platform. 1351 genes not detected in L1000 + inferred, include genes involved in mitochondria function, tretinoin and doxorubicin response and EZH2 regulated genes, and in particular genes in 9q34. This highlights that there are genes that are completely missed by L1000 and inference that have important biological function and are useful for clustering compounds based on their transcriptional response. (**Supplementary Table IV and Supplementary Figure 7e**).

	# Genes in Gene Set (K)	# Genes in Overlap (k)	p-value	FDR q-value
GO_MITOCHONDRION	1633	91	2.86E-23	5.08E-19
GO_ENVELOPE	1090	67	1.65E-19	9.79E-16
MARTENS_TRETINOIN_RESPONSE_DN	841	57	1.16E-18	4.14E-15
GGGCGGR_SP1_Q6	2940	117	6.63E-18	1.96E-14
LU_EZH2_TARGETS_UP	295	33	1.59E-17	4.04E-14
GRAESSMANN_APOPTOSIS_BY_DOXORUBICIN_UP	1142	64	1.01E-16	2.00E-13
chr9q34	267	30	4.15E-16	7.38E-13

We carried out tSNE clustering for 2938 L1000 measured + inferred genes and 394 L1000 measured genes respectively. To address reviewer's question, we used K-means clustering to identify clusters in each tSNE plot and identified miscategorized compounds (in circles) when compared with clustering using the full set 4289 genes. K-means clustering showed that 11% (10/88) and 19% (17/88) compounds were miscategorized when using L1000 measured + inferred or only measured genes (**Supplementary Figure 7a-c**).

a. Using all 4289 genes

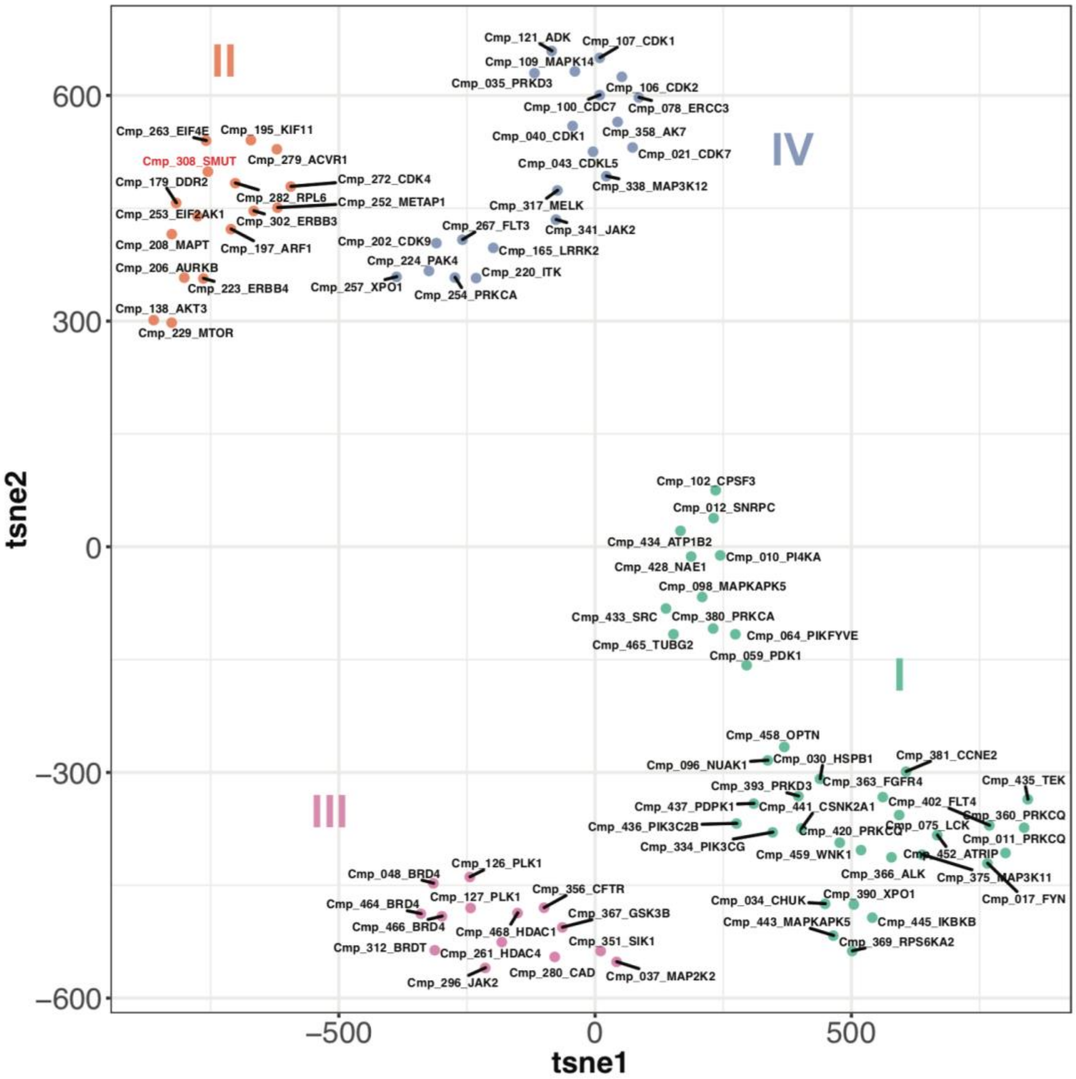

b. Using 2938 L1000 measured and inferred genes

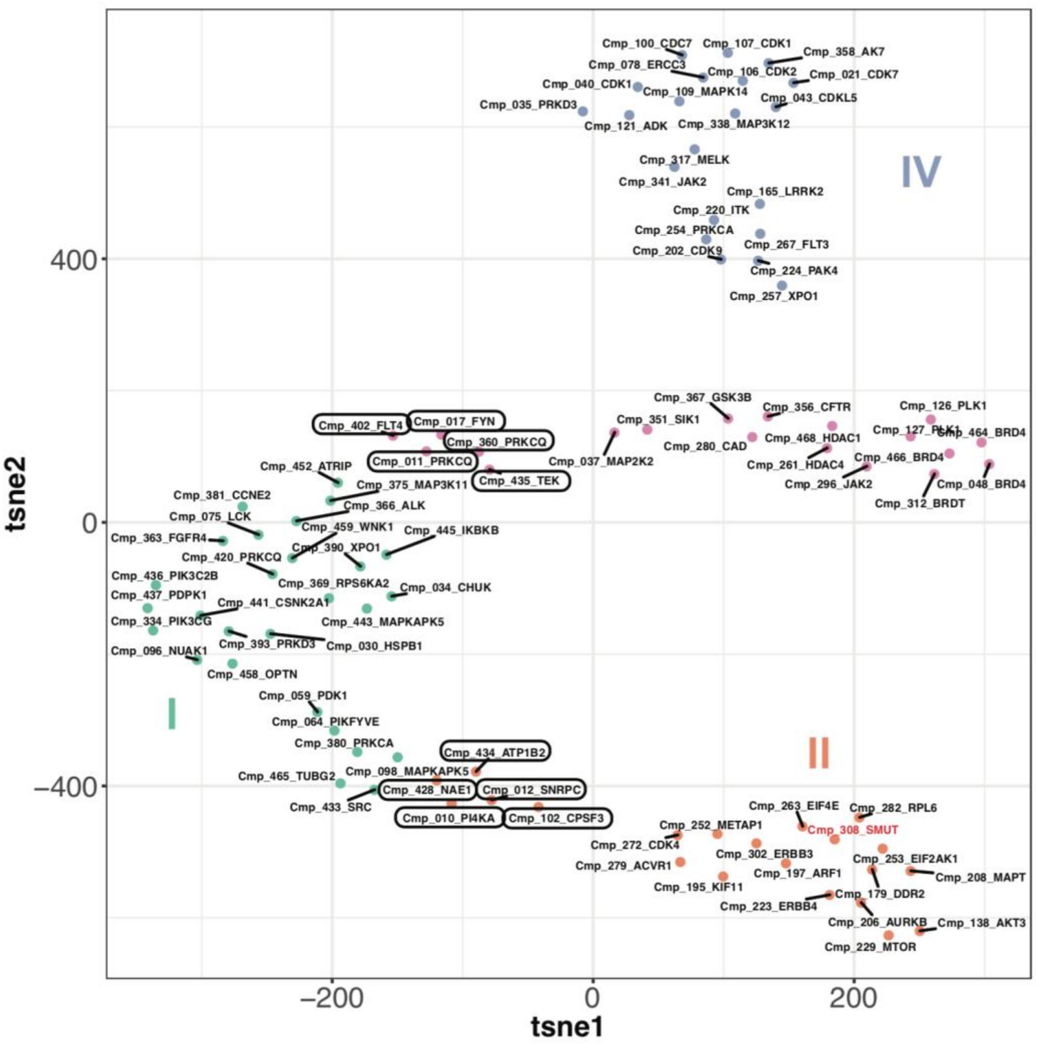

c. Using 394 L1000 measured genes

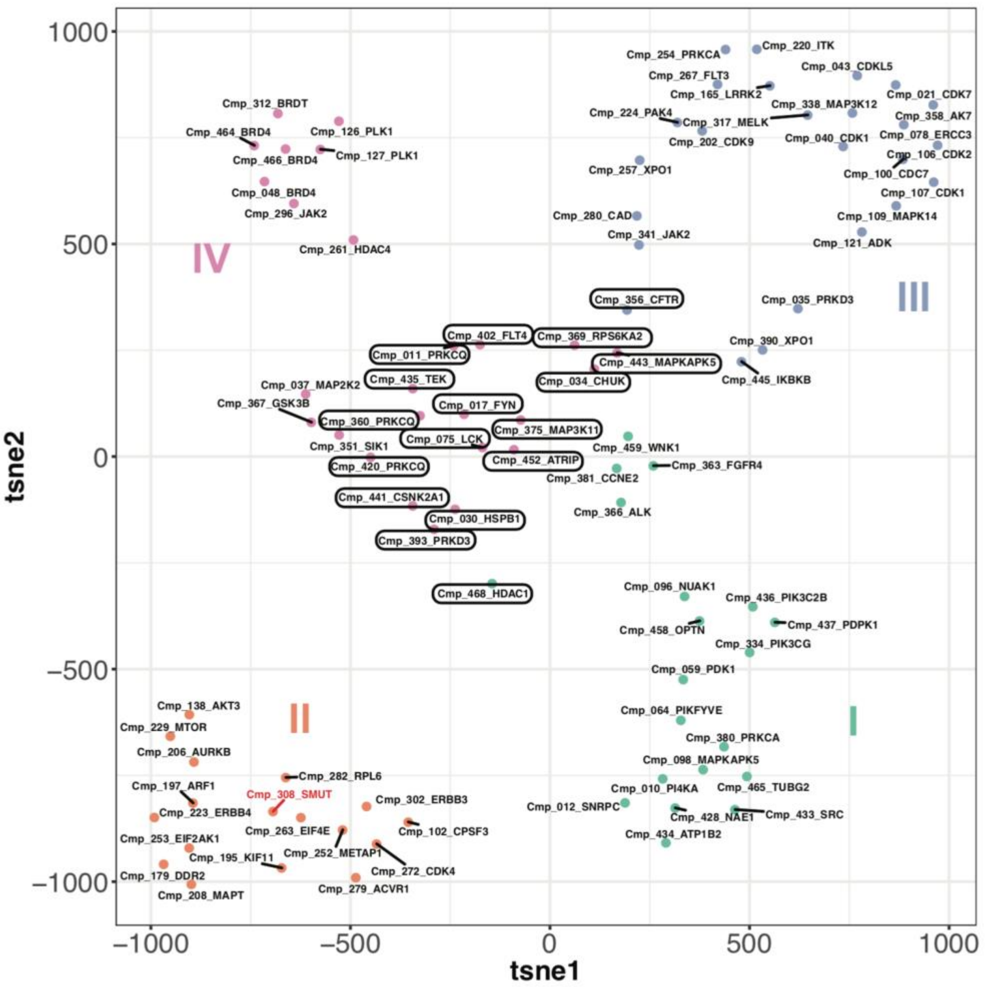

As a measurement of cluster separation, we calculated the ratio of mean distance of points within K-means clusters to the distance between centroids of clusters (**Supplementary Figure 7d**). There is increased inter-cluster distance to intro-cluster distance ratios when using L1000 genes, suggestion suboptimal separation of clusters.

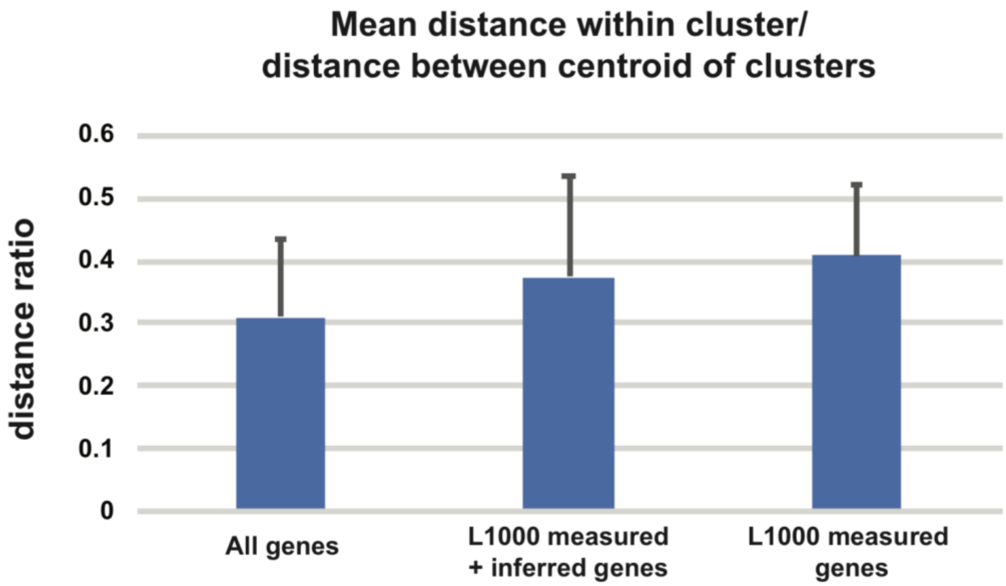

5. For Figure 4, it may be worth expanding on the utility of the compound vs. genetic perturbation. The RPL6 mRNA not dropping during compound treatment isn't surprising, so it was not clear what the takeaway is from the comparison. Are the additional areas considered off-target (i.e. side effects) or at least non-RPL6 mediated effects? I think clarification here could be helpful.

We appreciate the reviewer pointing this out. We included compound vs. genetic perturbation comparison because in pharmacological studies, genetic perturbation is often used for early target validation when specific compounds are not available. We set to address the differences as well as common effects between the two approaches given the advantage of whole transcriptome profiling afforded by DRUG-seq. We made modifications in the main text (lines 161 to 190) to reflect this rationale.

6. In Figure 4, how is it possible to compare cyclohex rna seq pattern w/ rpl6 pattern based on volcano plots? It would be better to have PCA analysis or something else here; clustering would be best.

In **Figure 4**, volcano plots were used to demonstrate the effective CRISPR knockout on the target gene. To demonstrate the similar effect between cyclohex treatment and rpl6 CRISPR, we took the reviewer's advice and added **Supplementary Figure 6** to show clustering of samples. Treatment samples using compound or sgRNA clearly separate from control samples using DMSO or Non-targeting control guides.

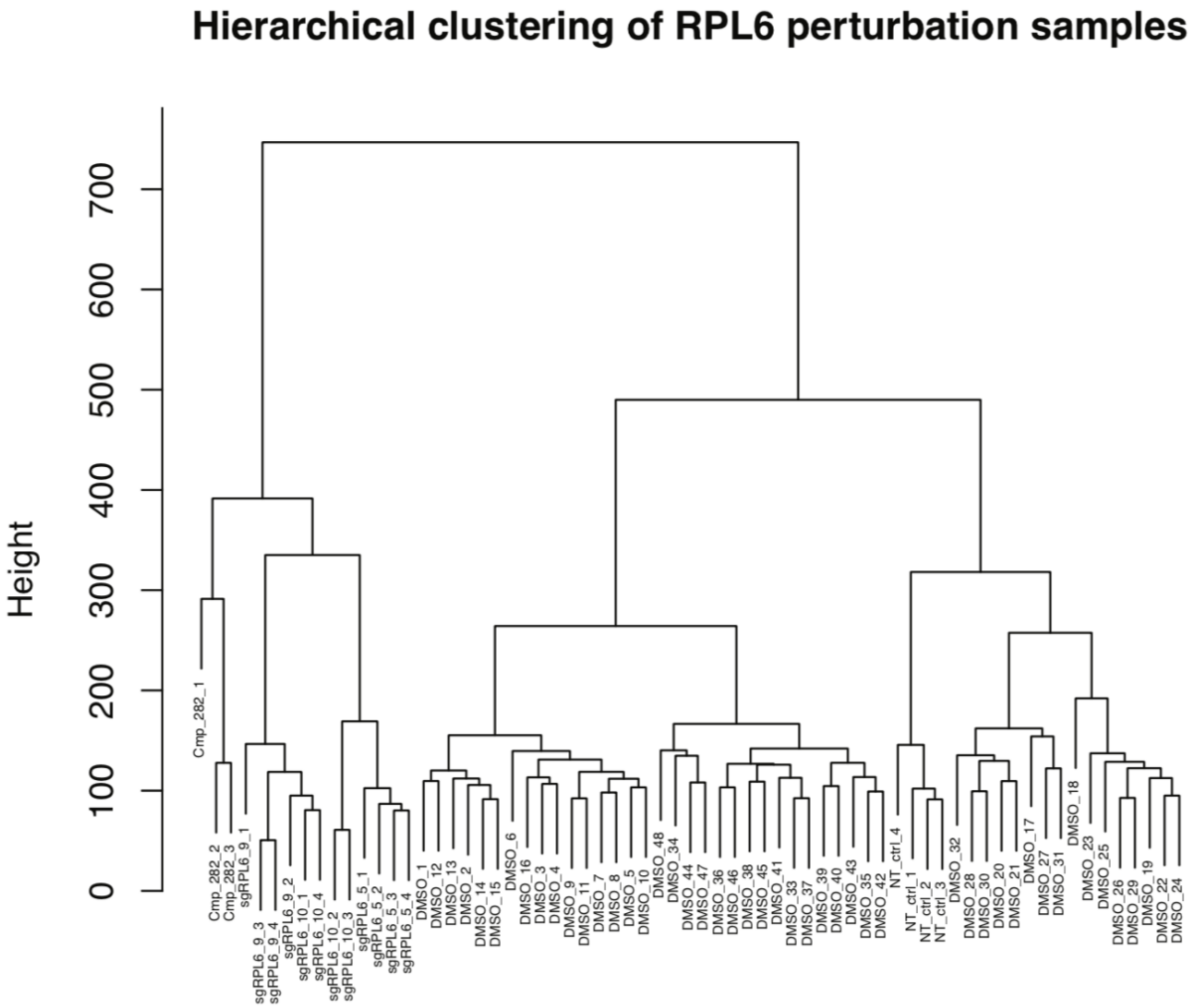

7. For the CRISPR experiment, how long were the cells given to generate indels? Was the Cmp_282 treatment comparison in Figure 4 for the same amount of time or 12 hours as in the compound characterization? It is probably hard to synchronize the drug with the editing event, but if the times are different, it could be acknowledged as potential difference between the treatments. For example, it could be that the extra effects picked up by DRUG-Seq are acute but the CRISPR cells have adapted during the extended recovery period to not show those transcriptional effects.

As explained in methods, CRISPR samples were collected at day 4. Cmp_282 treatment was carried out for 12 hours. We agree with the reviewer that this could be one of the reasons to explain the differences between compound effect and CRISPR and made changes in the main text (lines 161 to 190).

Minor comments:
1. line122/123 (and throughout the manuscript really): it will be clearer in most cases to refer to compounds by name (here, brusatol)

In most cases, we modified the text to reflect the compounds' names when they were first mentioned. However, some compound such as Cmp_253 does not have a recognizable synonym, and we decide to keep the pseudo compound name.

2. line 125-6: "Together our result suggest that Nrf2 translation is Cap-dependent and requires EIF4E function and further defines the MOA of Cmp_308" is quite a stretch without more discussion of previous paper cited. It would be better to moderate claim and just suggest the association if there is not to be direct follow-up

We made appropriate changes to the main text (lines 123 to 137) and we appreciate the reviewer's suggestion.

3. line 161: for clarity, just refer to this compound as cycloheximide

Main was text modified to include cycloheximide.

4. Line 9; phrasing in abstract to 'screen all targets at once' is confusing. really this is measurement of RNAs that are perturbed rather than the target of the drug. writing clarity could be improved with a transition sentence in abstract describing the general notion that rnaseq can be used as a proxy for drug effect

We agree with the reviewer and made changes in the summary lines 9 and 10.

5. the authors claim of ability to detect nuanced perturbation differences from related drug molecules is not supported

We intended to illustrate that transcriptome wide profiling afforded by DRUG-seq allows detection of differences among compounds engaging the same target, which was demonstrated in the Venn-diagram in **Figure 3f**. However, we do agree that perturbation differences go beyond transcriptome, and DRUG-seq provides only one phenotypic readout. We've made adjustments in the **Summary** (lines 17 to 19).

6. choice of unknown molecules to begin with is odd and makes benchmarking difficult, e.g. in line 83, compounds (Cmp_078 and Cmp_263). what are these? where did they come from? why not start with e.g. taxol or something? in any case, some kind of description/justification of these choices is needed

Cmp_078 (triptolide) is a potent transcription inhibitor, and Cmp_263 (homoharringtonine) is a potent translation inhibitor. They're selected as representations of different mechanism for comparison. Modification has been made in the main text (lines 82 to 86).

7. The authors then proceed in Fig. 3 directly to a tsne plot, not really discussing which compounds are used for benchmarking...are these the known targets or the predictions from the analysis here?

Targets for all compounds in our assay are known and listed in **Supplementary Table III**. We explained in the main text (lines 113 to 115) that out of all the compounds we profiled, 88 potent

compounds have more than 50 genes significantly changed and were chosen for tsne plot. This is to show how transcription readout by DRUG-seq corroborated with the MoA derived from known targets of these compounds. And indeed, we observed distinct clustering consistent with common pathways indicated by the targets of compounds.

8. In fig. 2d, need to label all axes, dendrograms directly on panel

Figure 2d x axes is calculated distance between individual genes, and y axes are samples tested in each platform, as color coded on the right side.

Point-by-point rebuttal for Reviewer 2:

Reviewer 2
1. Figure 2b shows that RNA-seq is able to detect a larger number of genes with FPKM/UMI distribution 0-1 and 10-100, but unexpectedly there is not better detection of the other gene subgroups. Please explain. In particular, why would RNA-seq detect a smaller number of genes in the >100 category compared to 13mil/well DRUG-seq?

We reexamined the data and realized the size factor for DRUG-seq was not calculated correctly during normalization. We recalculated the average gene detection, and also standard deviation of gene detection among samples and remade **Figure2b**. Average transcript level between 0~1 gives population RNA-seq the most advantage of gene detection due to more starting materials for lowly expressed genes. The rate of RT reaction and strand switching in DRUG-seq may be limited by cellular factor interference in one pot reactions with direct cell lysate, which was observed most pronouncedly at 10~100 level, while in population RNA, the reaction rate is less limited by starting with purified mRNA and implementing purification steps along library construction process.

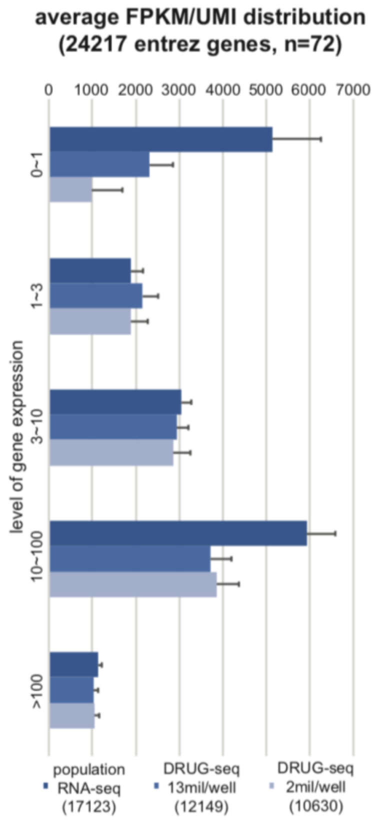

2. How consistent are the results (in terms of what genes are detected and their quantified expression levels) for DRUG-seq libraries sequenced at 2 million reads/well?

We constructed DRUG-seq libraries and compared 18 DMSO samples sequenced at 2 mil reads/well. Sample to sample correlation all exceed 0.9. When comparing randomly selected 2 samples, we detected highly consistent gene expression levels, except at very low level of gene expression, when gene expression itself becomes stochastic, and substrate recovery reaches technical limit of the platform. Main text (lines 96 to 98) has been modified to add this observation with **Supplementary Figure 4a** and **4b**.

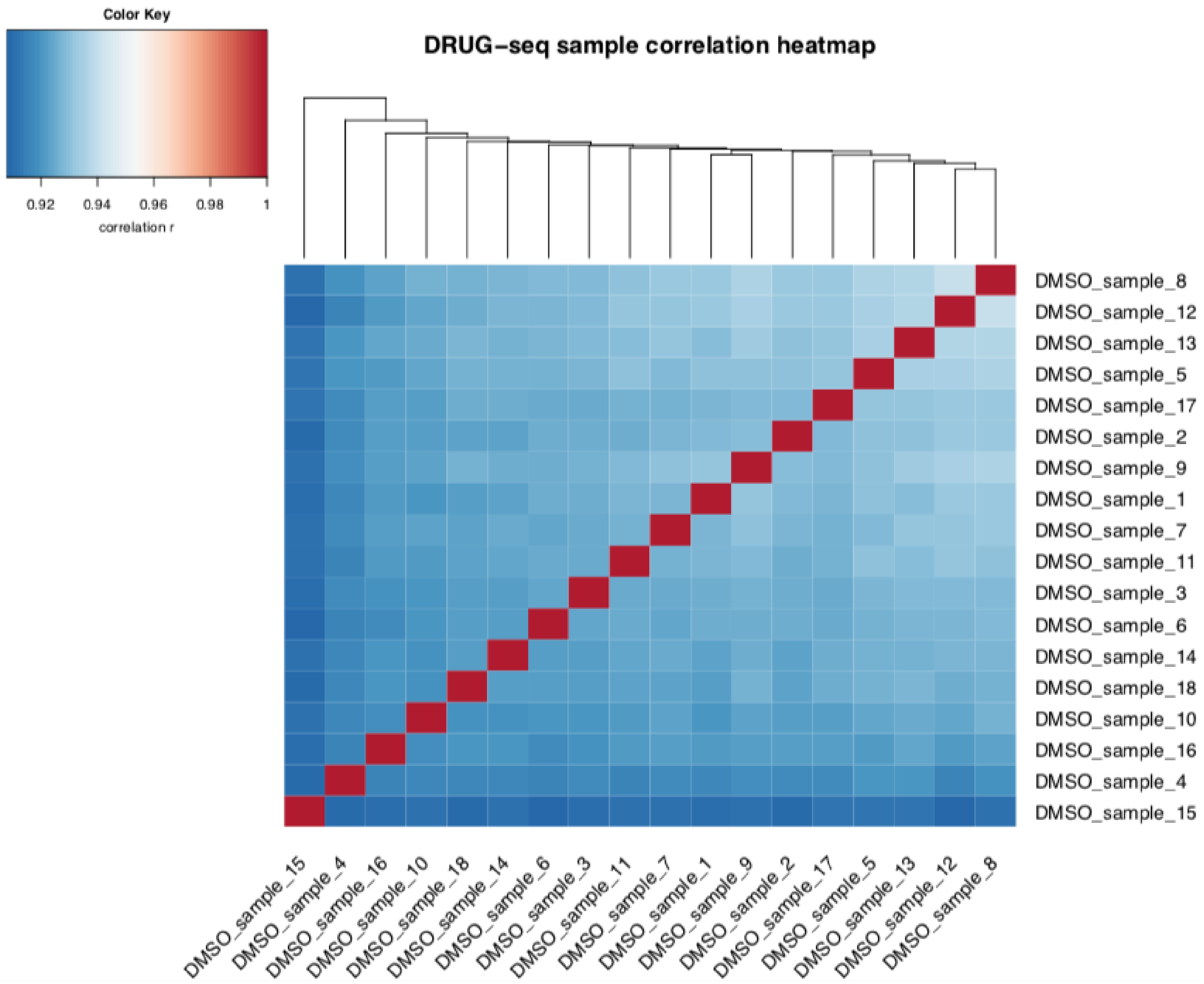
Color Key
0.92
0.94
0.96
0.98
1
correlation r
DRUG−seq sample correlation heatmap
DMSO_sample_8
DMSO_sample_12
DMSO_sample_13
DMSO_sample_5
DMSO_sample_17
DMSO_sample_2
DMSO_sample_9
DMSO_sample_1
DMSO_sample_7
DMSO_sample_11
DMSO_sample_3
DMSO_sample_6
DMSO_sample_14
DMSO_sample_18
DMSO_sample_10
DMSO_sample_16
DMSO_sample_4
DMSO_sample_15
DMSO_sample_15
DMSO_sample_4
DMSO_sample_16
DMSO_sample_10
DMSO_sample_18
DMSO_sample_14
DMSO_sample_6
DMSO_sample_3
DMSO_sample_11
DMSO_sample_7
DMSO_sample_1
DMSO_sample_9
DMSO_sample_2
DMSO_sample_17
DMSO_sample_5
DMSO_sample_13
DMSO_sample_12
DMSO_sample_8

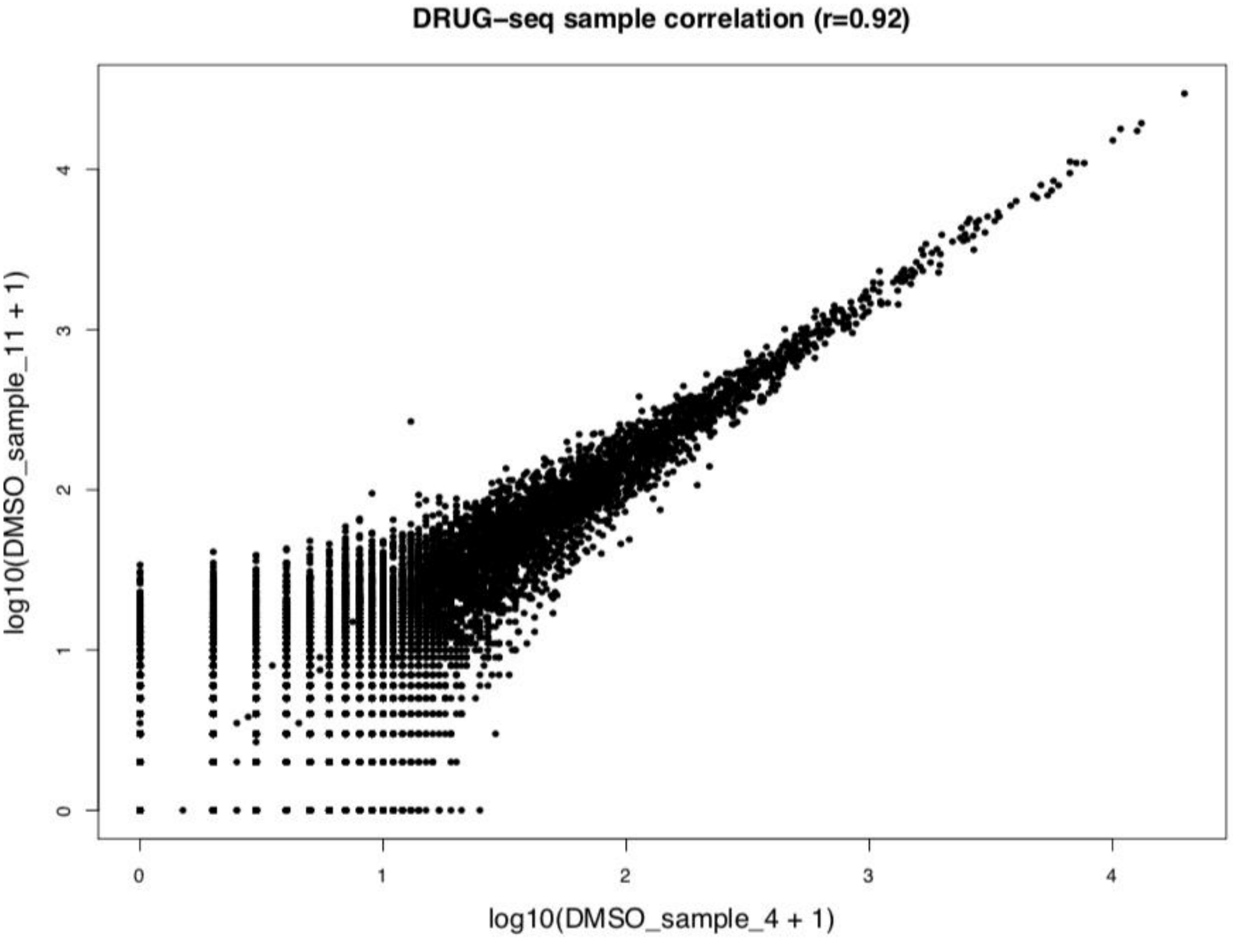

3. Only the 3' end of the transcripts are sequenced. Presumably this is a limitation due to the barcoding, but also this reduces the need for extensive sequencing. Some discussion should be addressed to consider the caveats of this approach. For example, for genes with similar 3' ends (such as pseudogenes, etc.), how does the limited sequencing impact alignment?

We appreciate the reviewer pointing out the advantage and limitations of the chemistry design, which is the same concept behind many other transcriptome library construction methods, such as CEL-seq, PLATE-seq, DROP-seq, inDrop, SPLiT-seq and commercial 10X genomics chromium single cell platforms. Genes with similar 3' end may be hard to discern using this chemistry, and care should be taken to ensure long enough read length for the transcripts. We identified 539 pseudo genes included in our entrez gene annotation. When comparing population RNA-seq and DRUG-seq sequenced at 2mil/well, we did not observe excessive under/over enrichment of these genes in DRUG-seq platform, suggesting consistency with population RNA-seq. The main text is modified (line 98-100 for added **Supplementary Figure 4c**.

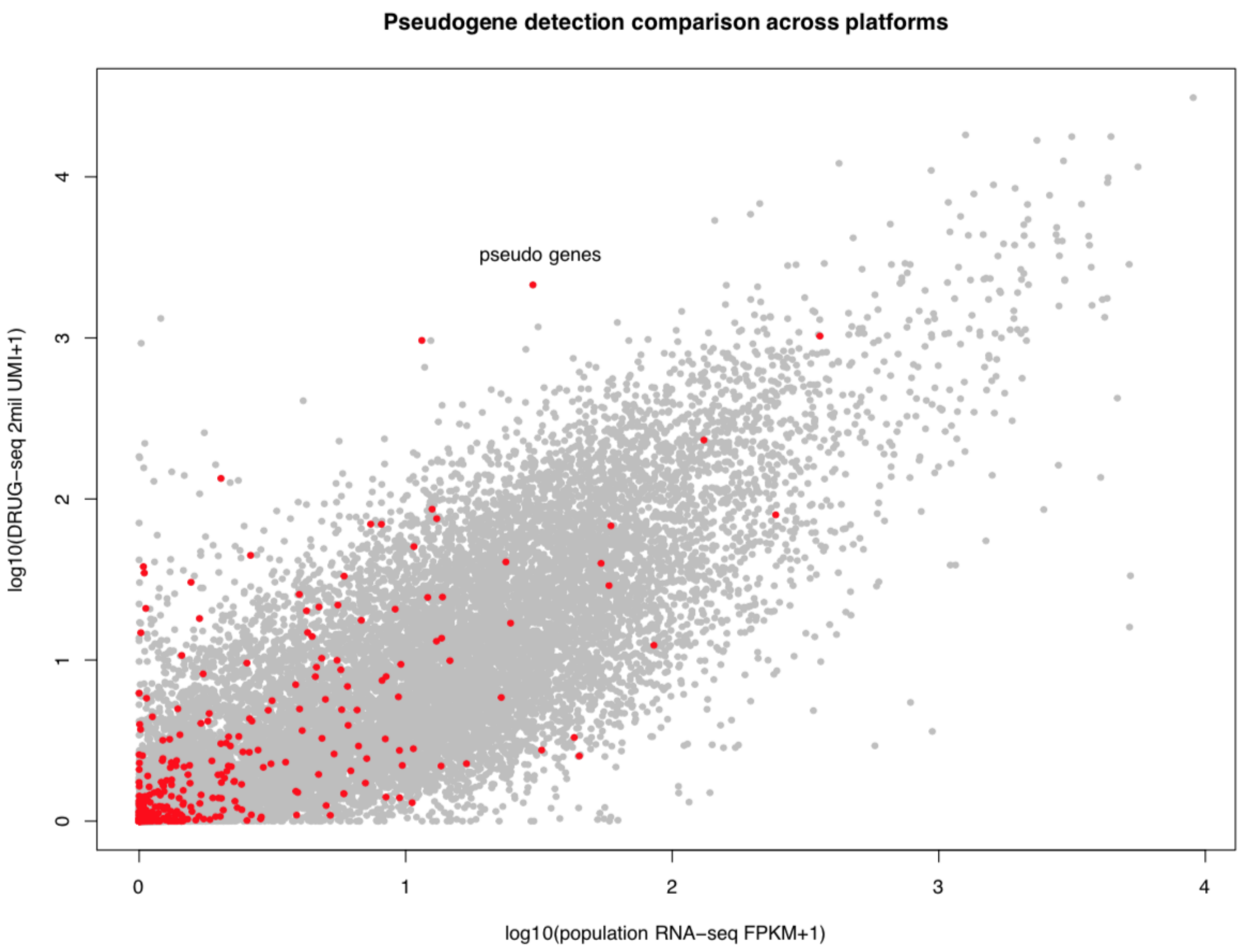

4. The use of DRUG-seq for the evaluation of CRISPR knock-outs is a reasonable idea, but the manuscript flow needs improvement. What is the motivation for testing RPL6 knockout? This sentence, "Unlike CRISPR treatment compound Cmp_282 didn't reduce RPL6 mRNA". What is the relevance of Cmp_282 in this section? Is it an inhibitor of RPL6?

CRISPR knock-out was compared to compound treatment of the same target because it is increasingly becoming a strategy for target validation during early discovery stage when target specific compounds are not available. Cmp_282 is cycloheximide, inhibitor of RPL6 and we modified the main text (lines 161 to 190) to make this clear. It is relevant that CRISPR reduces *RPL6* mRNA and Cmp282 does not because it highlights that CRISPR and compounds have different MOAs and this is partly responsible for differences in their transcriptional effects (line 187-190).

5. Since the 2mil/well DRUG-seq was able to capture almost the same information at L1000, it would be of interest to use the DRUG-seq data to approximate the dataset that would be collected by L1000, and then perform the clustering (Figure 3) to show whether DRUG-seq does provide superior clustering compared to methods that detect smaller numbers of transcripts.

L1000 assay measures only about 1000 genes, with computational algorithms to infer the expression of additional 10000 genes. In DRUG-seq, all gene expression levels were measured directly. When clustering DRUG-seq data, we selected up to 200 of differentially expressed genes ($p<0.05$ and $|\log2(\text{Fold Change})| >1$) for each compound to avoid dominating effect by compounds with many dis-regulated genes (see **Methods**), resulting in 4289 genes included in the tSNE clustering in **Figure 3a**. Of these, 394 are directly measured in L1000 platform, and 2938 are either measured or inferred in L1000 platform. 1351 genes not included in L1000 include genes involved in mitochondria function, tretinoin and doxorubicin response and EZH2 regulated genes, and in particular genes in 9q34 (**Supplementary Table IV and Supplementary Figure 7e**).

	# Genes in Gene Set (K)	# Genes in Overlap (k)	p-value	FDR q-value
GO_MITOCHONDRION	1633	91	2.86E-23	5.08E-19
GO_ENVELOPE	1090	67	1.65E-19	9.79E-16
MARTENS_TRETINOIN_RESPONSE_DN	841	57	1.16E-18	4.14E-15
GGGCGGR_SP1_Q6	2940	117	6.63E-18	1.96E-14
LU_EZH2_TARGETS_UP	295	33	1.59E-17	4.04E-14
GRAESSMANN_APOPTOSIS_BY_DOXORUBICIN_UP	1142	64	1.01E-16	2.00E-13
chr9q34	267	30	4.15E-16	7.38E-13

We carried out tSNE clustering for 2938 L1000 measured + inferred genes and 394 L1000 measured genes respectively. To address reviewer's question, we used K-means clustering to identify clusters in each tSNE plot and identified miscategorized compounds (in circles) when compared with clustering using the full set 4289 genes. K-means clustering showed that 11% (10/88) and 19% (17/88) compounds were miscategorized when using L1000 measured + inferred or only measured genes (**Supplementary Figure 7a-c**).

a. Using all 4289 genes

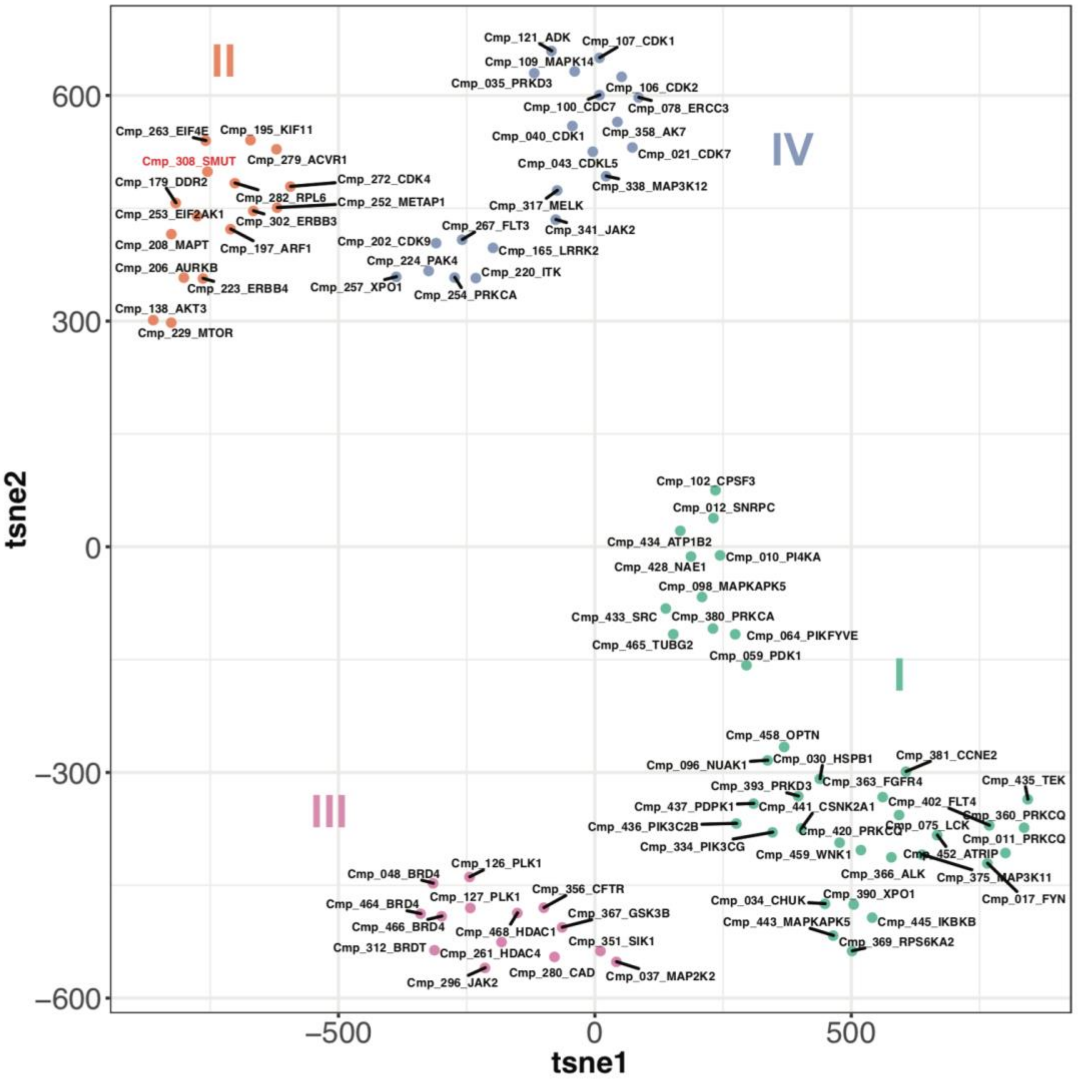

b. Using 2938 L1000 measured and inferred genes

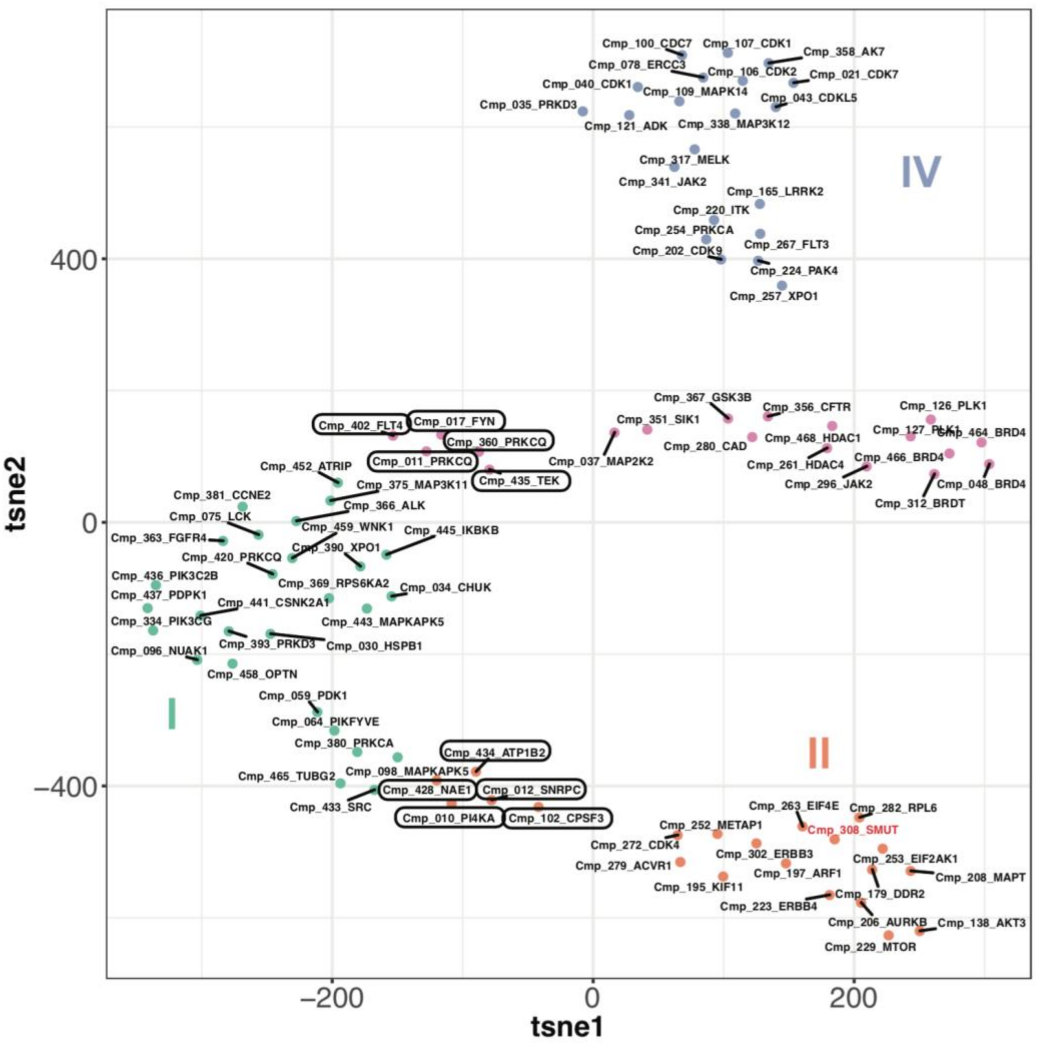

c. Using 394 L1000 measured genes

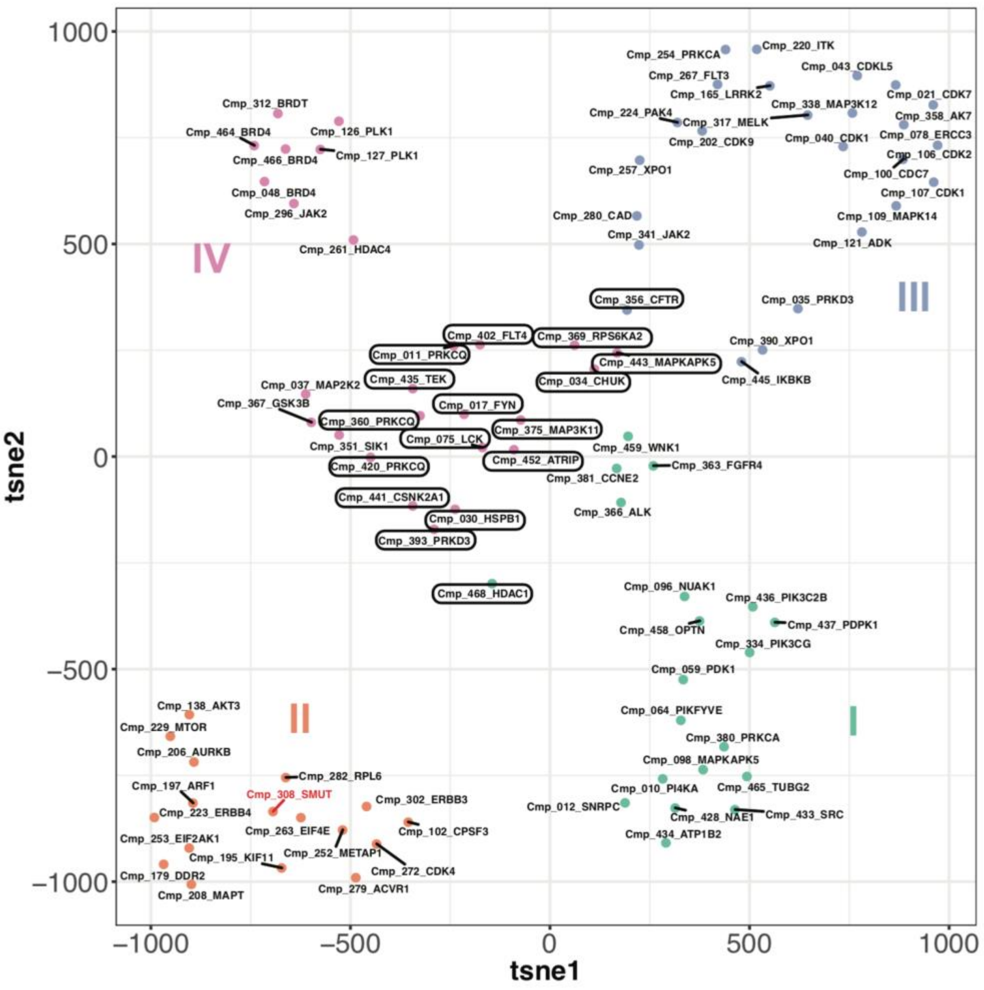

As a measurement of cluster separation, we calculated the ratio of mean distance of points within K-means clusters to the distance between centroids of clusters (**Supplementary Figure 7d**). There is increased inter-cluster distance to intro-cluster distance ratios when using L1000 genes, suggestion suboptimal separation of clusters.

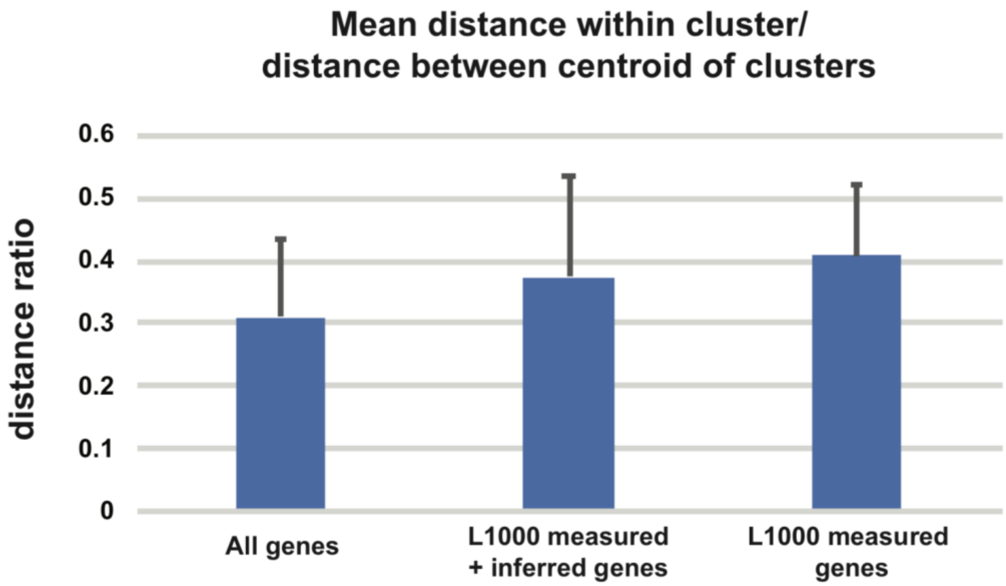

6. The information in the supplementary tables is cut off.

We checked the supplementary tables and the information seems intact. Please advise which specific table is cut off and we'll double check.
